# Identification of endoplasmic reticulum stress-related biomarkers of diabetes nephropathy based on bioinformatics and machine learning

**DOI:** 10.3389/fendo.2023.1206154

**Published:** 2023-09-01

**Authors:** Jiaming Su, Jing Peng, Lin Wang, Huidi Xie, Ying Zhou, Haimin Chen, Yang Shi, Yan Guo, Yicheng Zheng, Yuxin Guo, Zhaoxi Dong, Xianhui Zhang, Hongfang Liu

**Affiliations:** ^1^ Dongzhimen Hospital, Beijing University of Chinese Medicine, Beijing, China; ^2^ Key Laboratory of Chinese Internal Medicine of Ministry of Education and Beijing, Renal Research Institution of Beijing University of Chinese Medicine, Dongzhimen Hospital Affiliated to Beijing University of Chinese Medicine, Beijing, China

**Keywords:** diabetic nephropathy, endoplasmic reticulum stress, WGCNA (weighted gene co-expression network analysis), machine learning, immune cell infiltration, molecular subtypes

## Abstract

**Backgrounds:**

Diabetes nephropathy (DN) is a growing public health concern worldwide. Renal dysfunction impairment in DN is intimately linked to ER stress and its related signaling pathways. Nonetheless, the underlying mechanism and biomarkers for this function of ER stress in the DN remain unknown.

**Methods:**

Microarray datasets were retrieved from the Gene Expression Omnibus (GEO) database, and ER stress-related genes (ERSRGs) were downloaded from the MSigDB and GeneCards database. We identified hub ERSRGs for DN progression by intersecting ERSRGs with differentially expressed genes and significant genes in WGCNA, followed by a functional analysis. After analyzing hub ERSRGs with three machine learning techniques and taking the intersection, we did external validation as well as developed a DN diagnostic model based on the characteristic genes. Immune infiltration was performed using CIBERSORT. Moreover, patients with DN were then categorized using a consensus clustering approach. Eventually, the candidate ERSRGs-specific small-molecule compounds were defined by CMap.

**Results:**

Several biological pathways driving pathological injury of DN and disordered levels of immune infiltration were revealed in the DN microarray datasets and strongly related to deregulated ERSRGs by bioinformatics multi-chip integration. Moreover, CDKN1B, EGR1, FKBP5, GDF15, and MARCKS were identified as ER stress signature genes associated with DN by machine learning algorithms, demonstrating their potential as DN biomarkers.

**Conclusions:**

Our research sheds fresh light on the function of ER stress in DN pathophysiology and the development of early diagnostic and ER stress-related treatment targets in patients with DN.

## 1 Introduction

As a prevalent chronic microvascular complication of diabetes mellitus (DM), diabetic nephropathy (DN) is one of the primary causes of end-stage renal disease (ESRD), and the incidences of diabetes and DN have increased over the past decade (1). By 2045, it is anticipated that there will be 700 million diabetes individuals worldwide, of which 40% will develop DN (2). In terms of disability-adjusted life years (DALYs), the burden of DN is one-third of that of chronic kidney disease (CKD) (3). Consequently, DN is a huge global public health issue that causes a substantial burden on the economy and the health sector. The main clinical symptoms of DN are glomerular hyperfiltration, a gradual increase in urine albumin excretion rate, and a sustained decrease in glomerular filtration rate (GFR). Morphologically, early-stage DN is characterized by glomerular basement membrane thickening, mesangial matrix buildup, podocyte and tubular cell damage, and diffuse/nodular glomerulosclerosis and tubulointerstitial fibrosis with inflammation (4, 5). Currently, treatment for DN focuses on the primary disease and delays the disease’s progression. Controlling blood glucose and blood pressure, inhibiting the activation of RASS, sodium-glucose co-transporter 2 (SGLT2), strengthening exercise, improving diet, and reducing body weight are the primary means of preventing and treating DN (6, 7), which are still difficult to meet the clinical needs, with a large number of DN patients developing ESRD irreversibly. Therefore, it is imperative to investigate its pathogenesis further in order to identify effective biomarkers for early diagnosis and treatment.

With the ongoing investigation of the pathophysiological mechanism of DN, a potential emerging mechanism is the endoplasmic reticulum (ER), the location of protein folding and post-translational modifications and a key organelle of the secretory pathway (8). The ER does have a robust homeostasis system that can maintain the stability of its internal environment under normal conditions. The accumulation of unfolded or misfolded proteins in the lumen contributes to the pathological condition of ER dysfunction known as ER stress, which can be induced by a range of clinical conditions that affect ER homeostasis. Moderate ER stress is advantageous for repairing and stabilizing the intracellular environment, whereas prolonged ER stress can compromise ER function and induce apoptosis (9). According to a substantial body of evidence, the dysfunction of ER stress is associated with the onset and progression of DN. The kidney’s native cells (podocytes, tubular epithelial cells, endothelial cells, and mesangial cells) have a large and intricate ER system, which is a prerequisite for the formation of ER stress. Hyperglycemia, proteinuria, advanced glycation end products (AGEs), and free fatty acids (FFA) have been identified as important inducers of ER stress and its downstream signaling cascade activation in diabetic kidneys (10). Frequently, excessive ER stress develops in the renal intrinsic cells of DM patients, which can lead to cell damage, apoptosis and finally the formation of DN (11). Notably, ER stress inhibitors have been shown to enhance ER folding capacity, decrease ER stress, and halt the progression of DN (12). Nonetheless, a number of studies demonstrated that activated ER stress has a protective effect on DN, which reflects the bidirectional control of ER stress in DN (13). Consequently, a comprehensive analysis of ER stress should aid in the elucidation of the molecular mechanisms underlying the pathophysiology of DN and the expansion of the repertoire of potential diagnostic biomarkers.

Currently, research on DN has reached the gene level, and bioinformatics tools are routinely employed in the quest for prognostic or predictive biomarkers (14). Due to the lack of bioanalyses performed specifically for ER stress in DN to date, the purpose of this study was to use bioinformatics multi-chip analysis and the WGCNA algorithm to identify ER stress-related genes (ERSRGs) in renal tissues of DN patients using the gene sequencing chip of DN patients from the GEO database, and to combine machine learning algorithms to select characteristic genes. In addition, the related genes’ particular enrichment pathway and immune infiltration mechanism were studied. Finally, we hypothesized potential molecular subtypes linked with ER stress in DN patients and compared their immunological characteristics and molecular mechanisms. So as to fully understand the molecular mechanism underlying the ES stress-related pathophysiological process of DN and to discover appropriate biomarkers, thus providing a theoretical reference and theoretical underpinnings for early detection and targeted treatment of DN.

## 2 Materials and methods

### 2.1 Acquirement and processing of multi-chip dataset

The GEO database (http://www.ncbi.nlm.nih.gov/GEO) was accessed in order to retrieve DN microarray datasets. There were a total of five glomerular DN datasets (GSE30122, GSE47185, GSE96804, GSE99340, and GSE104948), one tubulointerstitial DN dataset (GSE104954), and one kidney biopsy DN dataset (GSE142025) downloaded, with the specifics of each dataset shown in **Table 1** and **Supplementary Table 1**. Each cohort’s probe ID was initially annotated and converted to “Entrez ID” according to platform annotation documents. Within five glomerular DN datasets, R (version 4.2.0) was used to construct a multi-chip dataset containing 100 healthy controls and 90 DN patients. Then, the R package “SVA” (version 3.46.0) containing the “Combat” function was applied to the multi-chip dataset to correct the batch effects. Using principal component analysis (PCA) to evaluate if the batch effect has been abolished, with GSE104954 and GSE142025 serving as the validation data set at last.

**Table 1:** Dataset details.

Datasets	Platforms	Tissue	Citation(s)	DN patients	Healthy controls	Time
GSE30122	GPL571 (Affymetrix Human Genome U133A 2.0 Array)	Glomeruli	PMID: 21752957 PMID: 26190114	9	26	2011
GSE47185	GPL11670 (Affymetrix Human Genome U133 Plus 2.0 Array)	Glomeruli	PMID: 23950145	14	17	2013
GSE96804	GPL17586 (Affymetrix Human Transcriptome Array 2.0)	Glomeruli	PMID: 29242313 PMID: 30511699	41	20	2018
GSE99340	GPL19109 (Affymetrix Human Genome U133 Plus 2.0 Array) GPL19184 (Affymetrix Human Genome U133A Array)	Glomeruli	PMID: 29242313 PMID: 30511699	14	11	2017
GSE104948	GPL22945 (Affymetrix Human Genome U133 Plus 2.0 Array) GPL24120 (Affymetrix Human Genome U133A Array)	Glomeruli	PMID: 29724730	12	26	2018
GSE104954	GPL22945 (Affymetrix Human Genome U133 Plus 2.0 Array)	Tubulointerstitium	PMID: 29724730	17	18	2018
GSE142025	GPL20301 (Illumina HiSeq 4000)	Kidney biopsy	PMID: 31578193 PMID: 32086290	27	9	2019

### 2.2 Recognition of differentially expressed genes

Utilizing the R package “limma” (version 3.54.2) (15), a differential expression analysis was conducted between the DN and control groups using the multi-chip dataset. Then, genes with a |log-fold change (FC)| > 0.5 and a Benjamini and Hochberg adjusted with *p*-value < 0.05 were selected as differentially expressed genes (DEGs).

### 2.3 Functional annotation and enrichment analysis of DEGs

To further illuminate the biofunction of the selected DEGs, Gene Ontology (GO) enrichment analysis, Kyoto Encyclopedia of Genes and Genomes (KEGG) pathway analysis, and Disease Ontology (DO) enrichment analyses were done on DEGs using “ClusterProfiler” (version 4.6.2) within R and then visualized using “ggplot2” (version 3.4.2). And the biological process (BP), cellular component (CC), molecular function (MF), signal pathway, and disease type were screened with a threshold of *p*-value < 0.05. In addition, gene set enrichment analysis (GSEA) was utilized to determine the functional words that were most significant between the DN and control groups (16). The hallmark gene sets “c5.go.v7.4.symbols.gmt” and “c2.cp.kegg.v7.4.symbols.gmt” were obtained from the Molecular Signatures Database (MSigDB). These gene sets summarize and represent well-defined biological states or processes and have consistent expression. To obtain a normalized enrichment score for each analysis, 1000 times gene set permutations were performed. After 1000 permutations, a false discovery rate (FDR) < 0.25 and *p*-value < 0.05 was considered highly enriched.

### 2.4 Weighted gene co-expression network analysis

Weighted gene co-expression network analysis (WGCNA) is a system biology technique used to examine gene connection patterns between samples. It can be used to identify highly synergistic gene expression matrices, and to identify candidate core genes based on the interconnectedness of expression matrices and the degree of association between genes and phenotypes (17). In the current investigation, the “WGCNA” (version 1.72-1) function within R was used to discover related gene modules and to screen gene sets that may be especially associated with glomerular damage in DN. The module analysis soft threshold β is obtained by assessing the scale independence and average connectivity of modules under various weighted coefficients. After determining the soft threshold β, the scale-free topological distribution network was constructed, and the correlation matrix was transformed according to the pearson correlation coefficient between genes into the adjacency matrix, then into the topological overlap matrix (TOM) to obtain the differences between genes (1-TOM). In addition, the modules incorporated hierarchical clustering and dynamic tree cutting function detection. To classify genes with similar expression profiles into gene modules, the average linkage hierarchical clustering of the gene tree was performed using a “TOMbased” difference measurement method with a minimum genome size of 50, and the module membership (MM, correlation between specific genes and module characteristic genes) and gene significance (GS, correlation between specific genes and clinical variables) were calculated. Finally, the network of feature genes were shown.

### 2.5 Identification and enrichment analysis of hub ERSRGs

First, we extracted 1406 ERSRGs from the MSigDB (GOBP response to endoplasmic reticulum stress and GOBP regulation of response to endoplasmic reticulum stress) and GeneCards database (with relevance scores ≥ 10) (18), which are exhaustive datasets that curate ERSRGs from research articles (**Supplementary Table 2**). Second, we intersected these ERSRGs with the DEGs derived from the multi-chip dataset and genes in the major modules of WGCNA to obtain the gene expression profile of hub ERSRGs. Using the “pheatmap” (version 1.0.12) R package, the heat map depicting the expression of hub ERSRGs was created. As previously noted, GO enrichment analysis and KEGG pathway analysis were performed using the “ClusterProfiler” tool to show the biofunction of hub ERSRGs.

### 2.6 Integrating multiple machine learning algorithms to identify characteristic genes

The discovery of biomarkers has made extensive use of machine learning algorithms, which can produce more detailed models. Three machine learning classifiers were used to filter feature genes in the current study: least absolute shrinkage and selection operator (LASSO), support vector machine recursive feature elimination (SVM-RFE), and random forest classifier (RF) (19). Using the “cv.glmnet” function in the R package “glmnet” (version 4.1-7), a LASSO regression prediction model was developed that could fit the generalized linear model, while variables were filtered and complexity was simultaneously changed. The R package “e1071” (version 1.7-13) was used to do SVM-RFE, the 10-fold cross-validation algorithm was used as the resampling method for SVM-RFE, and the features were sorted by recursion. Moreover, using the “RandomForest” (version 4.7-1.1) function, a RF analysis was conducted, and decision tree classifier models were configured to score the classification variables iteratively, thereby finding features with high classification accuracy. The genes within the intersection of three subsets were then selected for further study as characteristic genes.

At last, to verify the selected intersecting genes reliability, gradient boosting decision tree (GBDT) model was used as well. GBDT is one of boosting methods, which uses the method of gradient boosting to carry on each iteration and finally builds a strong model (20). And the algorithm often needs to generate a certain number of decision trees to achieve the accuracy of satisfaction. So we used GBDT based on recursive feature elimination with 10-fold cross-validation to test our result in above three models. This part was carried out in Python (version3.9).

### 2.7 Validation of characteristic genes

A co-expression pattern network diagram was developed using the R package “corrplot” (version 0.92) based on the correlation between gene expression levels in order to elucidate the interaction between the characteristic genes. Besides, the tubulointerstitium (GSE104954) and kidney biopsy (GSE142025) datasets were used for external validation of the ability of the characteristic genes to differentiate DN from healthy control. Using the unpaired t-test, it was determined if there was a difference in the expression of specific genes between the two groups at *p*-value < 0.05.

### 2.8 Construction and validation of a nomogram

The R package “rms” (version 6.6-0) was used to combine a total of the characteristic genes into a logistic regression (LR) model, which was displayed as a nomogram. The area under the receiver operating characteristic (ROC) curve (AUC) was recognized as the quantitative evaluation criterion for determining the discrimination capacity of each characteristic gene and the nomogram. The ROC analysis was performed using the R package “pROC” (version 1.18.0) (21).

### 2.9 CIBERSORT immune cell infiltration analysis

We used the CIBERSORT method to identify the ratios of various immune cells in multi-chip data sets of DN and control groups and to depict the abundance of immune cells based on the gene expression matrix of multi-chip datasets. The “corrplot” program was used to construct the heat map illustrating the quantitative relationship between distinct immune cells, and *p*-value < 0.05 indicates a statistically significant difference between the two groups. In addition, “ggplot2” was employed to analyze the correlation between the expression of characteristic genes and the proportions of immune cells.

### 2.10 Unsupervised clustering of ER stress-related genes

Using the k-means algorithm of unsupervised clustering analysis (“ConsensusClusterPlus” R package version 1.62.0) (22), we classified 90 DN samples into distinct clusters based on the expression level of the characteristic genes with a total of 1,000 iterations. The best number of categories was found through cumulative distribution function (CDF) curves, a consensus matrix, and a cluster score that was greater than 0.90. Then, PCA analysis was done to illustrate the distributional distinction between immune subtypes, which was shown graphically.

### 2.11 Gene set variation analysis

GSVA, which is a non-parametric and unsupervised method for assessing the variation of gene set enrichment across a sample population (23), is utilized to elucidate the molecular mechanisms features between various ER stress subtypes. As reference sets, “c5.go.v7.4.symbols.gmt” and “c2.cp.kegg.v7.4.symbols.gmt” were selected from the MSigDB. The R package “GSVA” (version 1.46.0) and the “ssGSEA” function were utilized to determine the GSVA score for each gene set. The GSVA score indicates the enrichment of each genome in absolute terms. And the “limma” package was utilized to compare differences in GSVA score between subtypes for each genome with a threshold of *p*-value < 0.05.

### 2.12 CMap analysis

The CMap (https://portals.broadinstitute.org/cmap) is a public database that links diseases, genes, and medications based on similar or opposite gene expression profiles (24). It was utilized to identify prospective DN-targeting therapies. Upregulation and downregulation ERSRGs were translated into chip-specific probe sets for querying the CMap. Using norm cs and FDR, we created a list of CMap instances predicted to invert ERSRGs.

### 2.13 Statistical analysis

Computer is running the Windows 10 64-bit system, with Intel Core i5-13500H processor and RTX 4050 graphics card, and the highest Rui frequency is 4.7 GHz. All data calculations and statistical analysis were performed with the R (version 4.2.0) or Origin (version 9.1) software. ROC curves and AUC values were utilized to assess the predictive performance of the diagnostic model. Pearson’s analysis was used to conduct correlation analysis. In addition, an unpaired t-test was utilized to assess the differential expression levels of the DN-specific genes. All *p*-values were bilateral, and *p*-value < 0.05 was deemed statistically significant.

## 3 Results

### 3.1 DEGs identification and functional enrichment analysis

**Figure 1:**
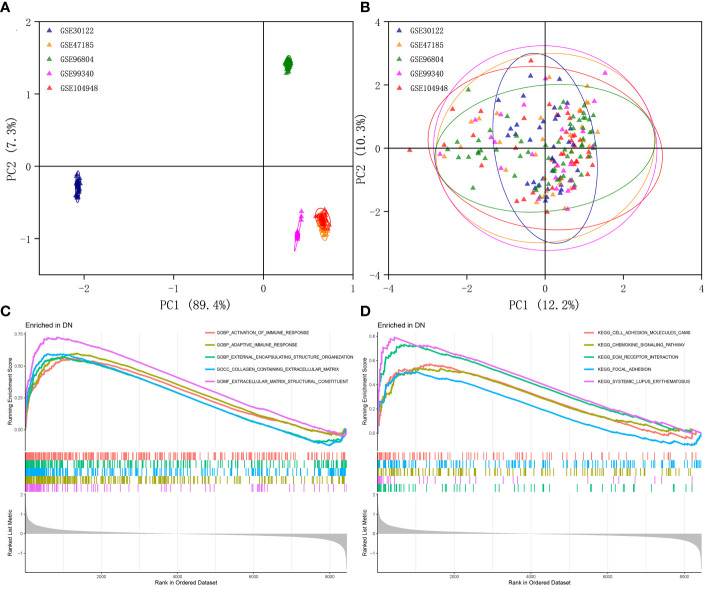
Identification of DEGs in glomeruli of DN patients. **(A)** Gene expression profiles without the removal of the batch effect. **(B)** Gene expression profiles with removal of batch effect. **(C)** Active GO functional enrichment of gene expression matrix in DN. **(D)** Active KEGG pathway of gene expression matrix in DN.

The multi-chip dataset included 100 healthy controls and 90 DN patients. After batch correction, PCA analysis revealed that the data distribution of each data set tended to be uniform (**Figures 1A, B**), implying that normalization was likely done properly. According to the screening criterion (|logFC| > 0.5, *p*-value < 0.05), 497 DN-specific related DEGs were found across five databases, 242 of which were downregulated and 255 of which were upregulated compared to healthy controls (**Supplementary Figure 1A**).

Further functional enrichment analysis uncovered the following GO terms to be the most significantly enriched: BP: urogenital system development; CC: collagen-containing extracellular matrix; MF: glycosaminoglycan binding (**Supplementary Figure 1B**). Further significantly enriched KEGG pathways were screened: PI3K-Akt signaling pathway, complement and coagulation cascades, focal adhesion, phagosome, AGE-RAGE signaling pathway in diabetic complications, protein digestion and absorption, ECM-receptor interaction, and renin-angiotensin system et al. (**Supplementary Figure 1C**). DO analysis revealed that majority of the genes were involved in urinary system and kidney diseases (**Supplementary Figure 1D**). In addition, the GSEA results demonstrated that in the renal tissue gene expression matrix of DN patients, active GO functions are mainly enriched in biological processes including activation of immune response, adaptive immune response, and external encapsulating structure organization and extracellular matrix-related functions (**Figure 1C**). The active KEGG pathway were mainly enriched in cell adhesion molecules cams, chemokine signaling pathway, ECM receptor interaction, and focal adhesion (**Figure 1D**).

### 3.2 Weighted gene co-expression network construction

**Figure 2:**
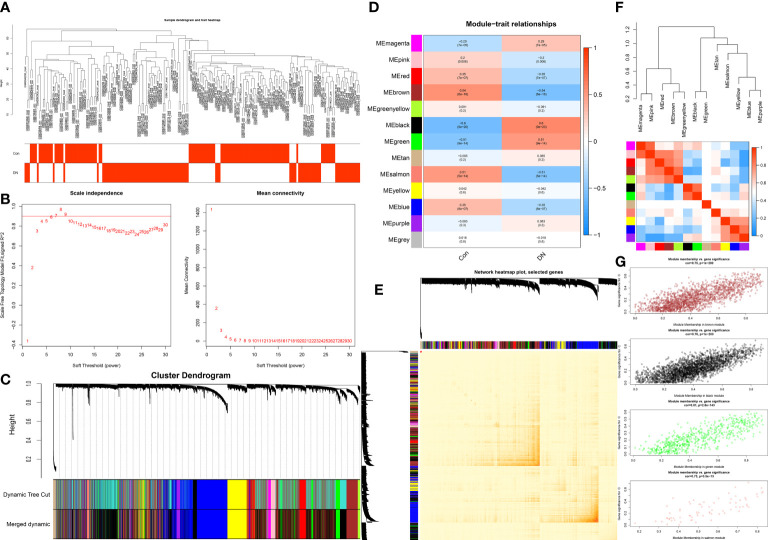
Construction of WGCNA co-expression network. **(A)** Sample clustering dendrogram with tree leaves corresponding to individual samples. **(B)** The screen of the best soft thresholds. Six was considered the best soft threshold. **(C)** The merging of similar modules. **(D)** Correlations between different modules and clinical traits. Red represents a positive correlation, and blue represents a negative correlation. **(E)** Clustering dendrogram of module feature genes. **(F)** Collinear heat map of module feature genes. Red color indicates a high correlation, blue color indicates opposite results. **(G)** The significance of genes related to DN in the brown, black, green and salmon module (a dot represents the genes in the module).

This work did a WGCNA analysis on the multi-chip dataset, and 100 healthy controls and 90 DN samples were chosen to cluster the samples and remove the obviously aberrant samples by establishing a threshold, as depicted in **Figure 2A**. Then, as shown in **Figure 2B**, when R2 is greater than 0.90 and the average connectivity is high, we set the soft threshold to 6. After combining the strongly linked modules using a 0.25 clustering height restriction, 13 modules were chosen for further investigation and displayed beneath the clustering tree (**Figure 2C**). Using the frontal correlations between ME values and clinical characteristics, the relationship between modules and clinical symptoms was investigated. The brown module was positively correlated with control (r = 0.54, p = 6e-16) and negatively linked with DN (r = -0.54, p = 6e-16), and the salmon module was positively correlated with control (r = 0.51, p = 5e-14) and negatively linked with DN (r = -0.51, p = 5e-14), while the black module was negatively connected with control (r = -0.6, p = 9e-20) and positively correlated with DN (r = 0.6, p = 9e-20), and the green module was negatively connected with control (r = -0.51, p = 9e-14) and positively correlated with DN (r = 0.51, p = 9e-14) (**Figure 2D**). Transcriptional correlation study within modules validated the reliability of module delineation by revealing no meaningful relationship between modules (**Figure 2E**). The findings of an examination of the correlation between modules revealed that there was no significant relationship between them (**Figure 2F**). Clinically relevant modules have been discovered. The findings revealed that the brown, black, green, and salmon modules were strongly associated with DN (**Figure 2G**). All genes within the four modules were investigated in greater detail.

### 3.3 Identification and characterization of hub ERSRGs

**Figure 3:**
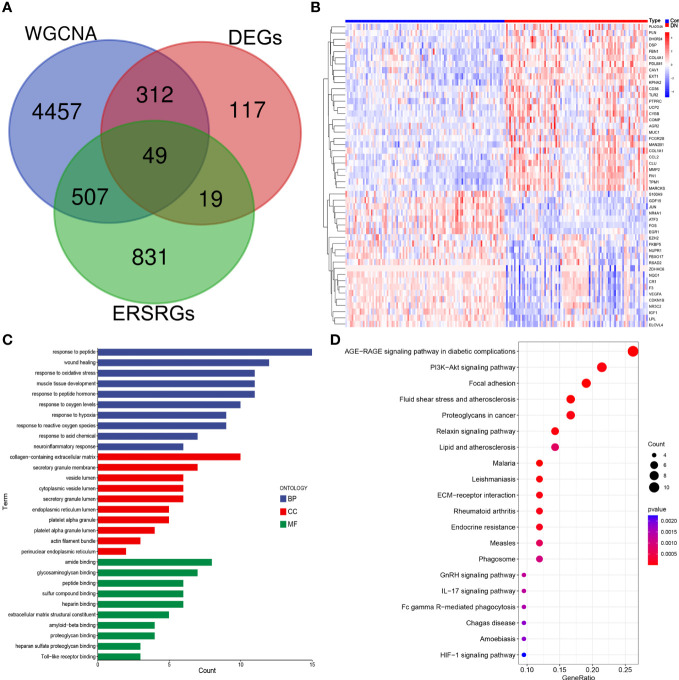
Identification of hub ERSRGs in the modules. **(A)** Venn diagram. **(B)** Heat map of hub ERSRGs. **(C)** GO enrichment analysis of hub ERSRGs. **(D)** KEGG enrichment analysis of hub ERSRGs.

After overlapping major module genes from WGCNA, DEGs, and ERSRGs using a Venn diagram, we discovered 49 overlapping genes (**Figure 3A**; **Supplementary Table 3**). A heat map was then created to illustrate the change and cluster relationship of hub ERSRGs (**Figure 3B**). In addition, we performed functional analysis in order to acquire a deeper comprehension of the biological functions of the hub ERSRGs. These genes were discovered to be linked with oxidative stress, collagen-containing extracellular matrix, endoplasmic reticulum lumen, extracellular matrix structural constituent, Toll-like receptor binding, and additional GO terms (**Figure 3C**). Further KEGG analysis showed that hub ERSRGs mainly participated in AGE-RAGE signaling pathway in diabetic complications, PI3K-Akt signaling pathway, focal adhesion, fluid shear stress and atherosclerosis, lipid and atherosclerosis, ECM-receptor interaction, endocrine resistance, phagosome, IL-17 signaling pathway, Fc gamma R-mediated phagocytosis, and HIF-1 signaling pathway et al. (**Figure 3D**).

### 3.4 Selection of characteristic genes *via* machine learning algorithm

**Figure 4:**
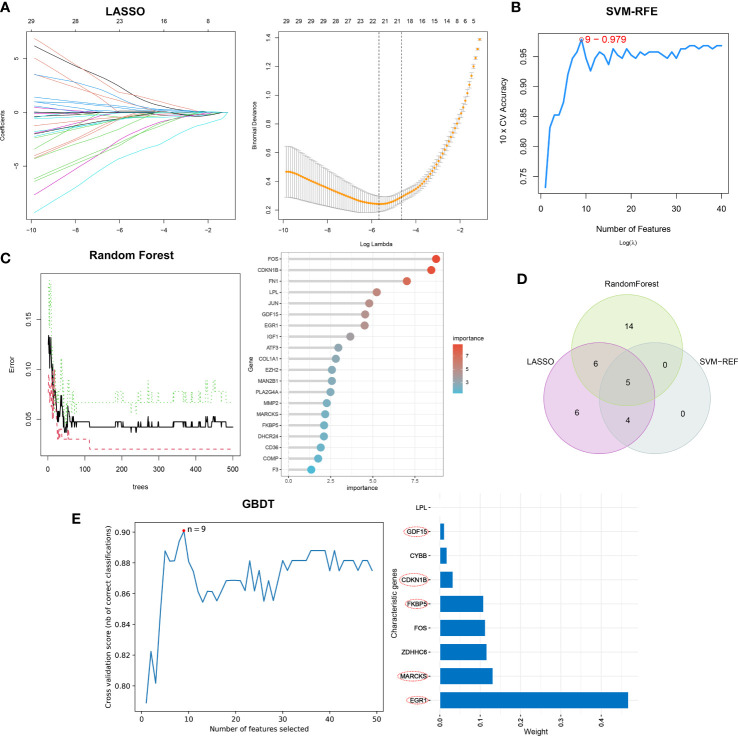
The selection of characteristic genes of DN via machine learning algorithm. **(A)** Twenty-one characteristic genes of LASSO. **(B)** Nine characteristic genes of SVM-RFE. **(C)** Top twenty characteristic genes of RF. **(D)** Visualization of intersecting genes. **(E)** GBDT to verified the selected intersecting genes reliability.

Separately, the LASSO, SVM-RFE, and RF algorithms were used to identify feature genes. When the construction of LASSO based on 10-fold cross-validation was applied, the minimal error value corresponded to twenty-one characteristic genes, including CCL2, CDKN1B, COL1A1, COL4A1, COMP, EGR1, ELOVL4, EXT1, FKBP5, FOS, GDF15, IGF1, KPNA2, LPL, MARCKS, NQO1, NUPR1, PLA2G4A, RSAD2, and S100A9 (**Figure 4A**; **Supplementary Table 4**). The SVM-RFE algorithm was validated by 10-fold cross-validation as well, and when the algorithm was most accurate and the estimation error was smallest, nine genes, including CCL2, CDKN1B, EGR1, FKBP5, GDF15, MARCKS, NQO1, PLA2G4A, and PLN, were determined (**Figure 4B**; **Supplementary Table 5**). RF in combination with feature selection was used to determine the association between error rate, number of classification trees, and the top 20 genes by weight were selected (**Figure 4C**). Consequently, five overlapping genes (CDKN1B, EGR1, FKBP5, GDF15, and MARCKS) were recognized based on the findings of above machine learning models (**Figure 4D**). Moreover, when testing the five overlapping genes reliability, these genes were selected by GBDT in the same way (**Figure 4E**).

### 3.5 Validation of characteristic genes expression and diagnostic capacity

**Figure 5:**
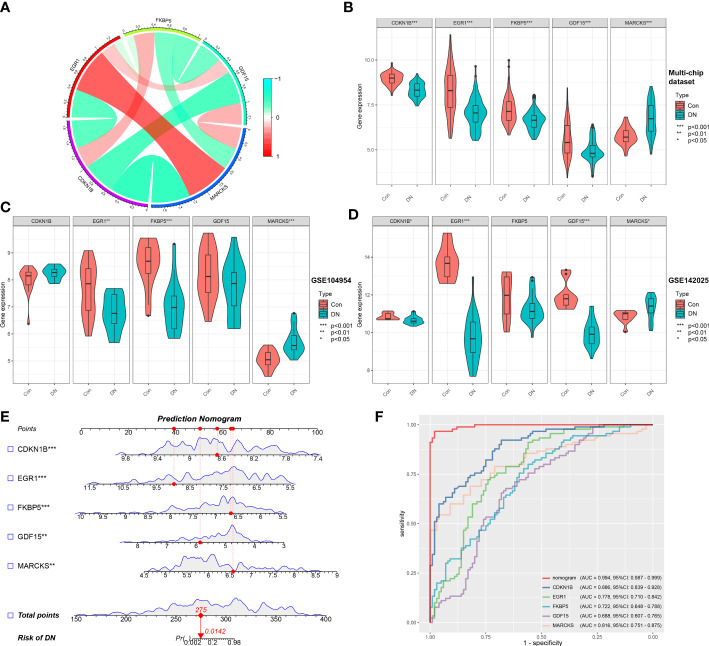
Validation of characteristic genes in the gene chip datasets. **(A)** Correlation analysis of characteristic genes. **(B)** Representative violin plots present the expression of characteristic genes in the multi-chip dataset. **(C)** Representative violin plots present the expression of characteristic genes in tubulointerstitium dataset (GSE104954). **(D)** Representative violin plots present the expression of characteristic genes in kidney biopsy dataset (GSE142025). **(E)** Prediction model of nomogram. **(F)** The ROC curves for evaluating the diagnostic performance. *p-value < 0.05; **p-value < 0.01; ***p-value < 0.001.

Based on the microarray expression matrix, the expression patterns of characteristic genes were investigated and validated. A heat map of co-expression correlations among the five genes were produced and displayed a significant interaction relationship (**Figure 5A**). Then we verified the expression of these five genes in the multi-chip dataset and found that CDKN1B, EGR1, FKBP5, and GDF15 expression was significantly lower in DN samples than in control samples, but MARCKS expression was significantly higher (**Figure 5B**). Significantly, gene expression patterns were found to be consistent between the tubulointerstitium (GSE104954) and kidney biopsy (GSE142025) testing cohorts (**Figures 5C, D**).

LR generated the prediction model based on the multi-chip dataset and displayed it as a nomogram with a c-index of 0.994 (**Figure 5E**), indicating a high correlation degree. Five genes identified in the multi-chip dataset (AUC > 0.6) exhibit diagnostic efficacy in differentiating DN patients from healthy controls, as confirmed by the ROC curve analysis of diagnostic power of characteristic genes (**Figure 5F**). In addition, external validation of nomogram in the validation data set (GSE104954 and GSE142025) showed that the c-index was 0.941 and 0.63, respectively (**Supplementary Figures 2A, B**). Notably, compare to single-core genetic models, the model of prediction nomogram had the best diagnostic performance. According to our findings, the aforementioned ER stress-related characteristic genes possessed a high diagnostic power and had the potential to act as diagnostic biomarkers for DN.

### 3.6 The results of immune cell infiltration

**Figure 6:**
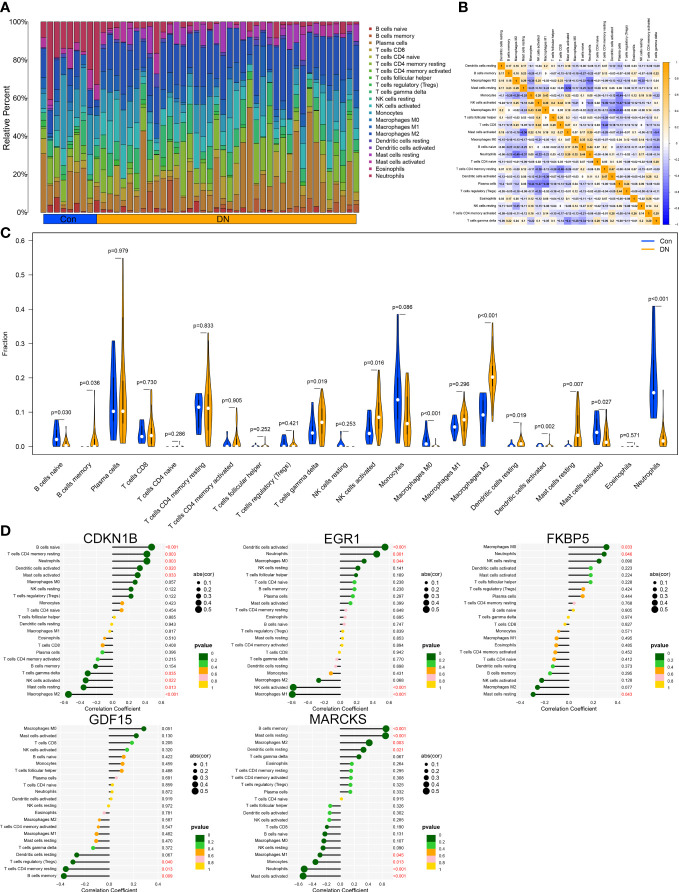
Immune cell infiltration analysis. **(A)** Distribution of 22 kinds of immune cells in tissues of DN and control groups. **(B)** Correlation diagram between immune cells. **(C)** Expression of immune cells in DN and control groups. **(D)** Immune cells correlation with the expression of characteristic genes.

Through the CIBERSORT method, we measured the characteristics of immunocytes between DN and control groups to further investigate the differential expression of immune components, and the cumulative histograms displayed the relative proportions of 22 immune cells (**Figure 6A**). The results indicated that activated Mast cells, activated Macrophages M2, activated Monocytes, activated NK cells, naive T cells CD4, and plasma cells constituted the majority. The correlation heat map for 22 immune cell types revealed a strong positive link between activated NK cells and Macrophages M1 and a significant negative correlation between activated Mast cells and resting Mast cells, Neutrophils and Macrophages M2, as well as Plasma cell and Macrophages M1 (**Figure 6B**). In addition, the violin plot regarding the difference in immune cell infiltration revealed significantly more B cells memory, T cells gamma delta, NK cells activated, Macrophages M2, Dendritic cells resting, and Mast cells resting in the experimental group than in the control group, whereas B cells naive, Macrophages M0, and Mast cells activated were significantly decreased (**Figure 6C**). Via analyzing immune cell correlations, we determined that specific genes may be implicated in the advancement of DN by controlling immune cells such as mast cells, neutrophils, and NK cells (**Figure 6D**).

### 3.7 Construction of ER stress subtypes in DN

**Figure 7:**
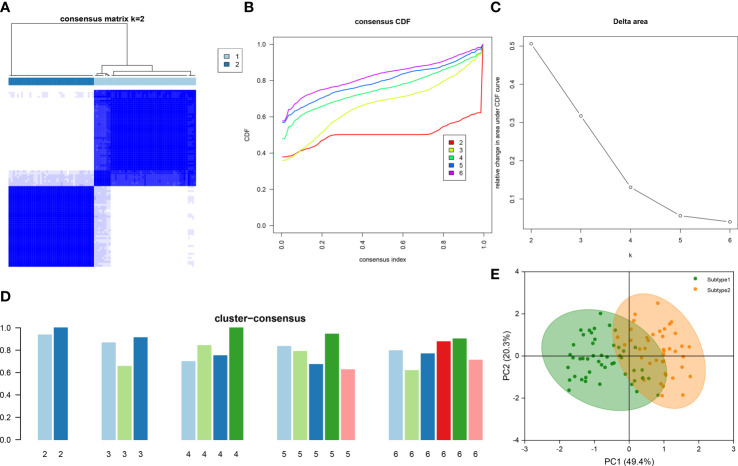
Identification of ER stress-associated molecular patterns in DN. **(A)** Consensus clustering matrix when k = 2. **(B)** Representative CDF curves when k = 2 to 6. **(C)** Relative alterations in CDF delta area curves. **(D)** Consensus score in each subtype when k = 2 to 6. **(E)** PCA analysis demonstrates that the DN patients are classified into two distinct subtypes.

To illustrate the ER stress-related patterns in DN, we designed a new consensus clustering approach to classify 90 DN samples based on the expression landscapes of five ER stress-related characteristic genes. The optimal number of subtypes was two, which was determined using a consensus matrix plot, a CDF plot, relative alterations in the area under the CDF curve, and consistent cluster score (> 0.9) (**Figures 7A–D**). We thus divided DN samples into two distinct subtypes, including subtype1 (n = 49) and subtype2 (n = 41). The PCA analysis demonstrated the striking distinction between the subtypes (**Figure 7E**).

### 3.8 Differentiation of immune characteristics and molecular mechanisms between ER stress subtypes

**Figure 8:**
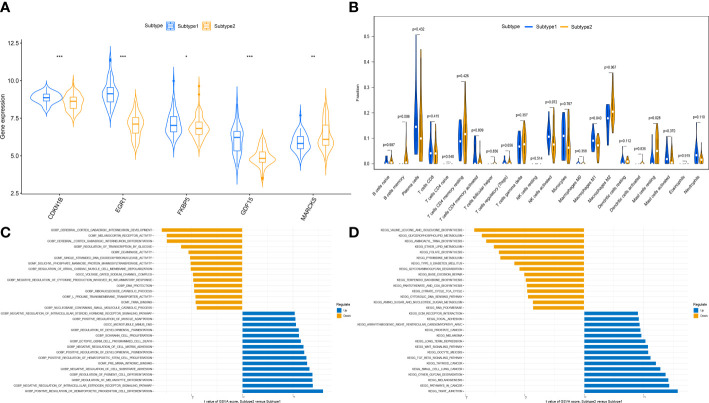
The different immune characteristics and molecular mechanisms between two subtypes. **(A)** Vio plots showing the mRNA expression of characteristic genes in two ER stress subtypes. *p-value < 0.05; **p-value < 0.01; ***p-value < 0.001. **(B)** Vio plots demonstrating the infiltration levels of immune cell components in two ER stress subtypes. **(C)** Differences in enriched biological functions between ER stress subtypes ranked by t value of GSVA score. **(D)** Differences in the enriched hallmark pathways between ER stress subtypes ranked by t value of GSVA score.

To clarify the molecular differences between these subtypes, the differential expression of characteristic genes among different ER stress-related subtypes was first evaluated. Subtype1 exhibited higher expression of CDKN1B, EGR1, FKBP5, and GDF15, whereas subtype2 was characterized by higher MARCKS expression (**Figure 8A**). However, there was no substantial difference in immune cell infiltration between the two subtypes (**Figure 8B**). Then, the GSVA analysis was conduct to evaluate the differences of molecular mechanisms in the subtypes with different ER stress expression patterns. The functional enrichment results suggested that negative regulation of cell substrate adhesion, positive regulation of hematopoietic stem cell proliferation, negative regulation of cell matrix adhesion, and negative regulation of intracellular steroid hormone receptor signaling pathway were prominently upregulated in subtype2 (**Figure 8C**). In addition, pathways enrichment results revealed that TGF-β signaling pathway, WNT signaling pathway, focal adhesion, and ECM receptor interaction were significantly elevated in subtype2 (**Figure 8D**). We determined that subtype2 is more closely related with ER stress in DN.

### 3.9 Results of CMap analysis

Moreover, we performed a CMap (http://portals.broadinstitute.org/cmap/) analysis to find potential drug candidates for DN to reverse the altered expression of hub ERSRGs. **Table 2** displays the top10 CMap compounds, with the protein synthesis inhibitor bruceantin having the highest negative connectivity score.

**Table 2:** CMap analysis indicated potential treatment options for DN.

Rank	CMap name	Moa	norm_cs	FDR
1	bruceantin	Protein synthesis inhibitor	-2.013	15.654
2	IKK-16	IKK inhibitor	-1.933	15.654
3	mycophenolate-mofetil	Inosine monophosphate dehydrogenase inhibitor|Dehydrogenase inhibitor|Hydroxycarboxylic acid receptor agonist|Immunosuppressant|Inositol monophosphatase inhibitor	-1.905	15.654
4	verrucarin-a	Protein synthesis inhibitor	-1.872	15.654
5	teroxirone	DNA inhibitor	-1.859	15.654
6	adapalene	Retinoid receptor agonist	-1.849	15.654
7	mitomycin-c	DNA alkylating agent|DNA inhibitor	-1.834	15.654
8	honokiol	AKT inhibitor	-1.833	15.654
9	ASC-J9	Androgen receptor agonist	-1.828	15.654
10	irinotecan	Topoisomerase inhibitor	-1.810	15.654

## 4 Discussion

DN is the most prominent type of CKD and the leading cause of ESRD in adults, accounting for 40% of patients requiring renal replacement therapy (25). Notwithstanding, the deficiency of existing diagnostic markers, the heterogeneity of pathogenesis and the lack of pathological diagnosis in clinical, which increases the difficulty of defining and comprehending of DN, causing a large number of patients have not achieved satisfactory results. Consequently, it is necessary to uncover more potent diagnostic markers and more appropriate molecular subtypes and to develop a diagnostic paradigm for DN.

ER stress refers to the aberrant structure and function of the ER as a result of many pathophysiological causes, including high glucose, hypoxia, oxidative/nitride stress, acidosis, calcium homeostasis imbalance, nutrient deficiency or excess, infection, etc. And therefore to block the protein processing and secretion are blocked in the ER, leading to an excessive accumulation of unfolded and misfolded proteins in the ER lumen (26). According to, the well studied unfolded protein response (UPR) is the primary signaling pathway of ER stress (27). UPR is mediated by three ER-resident sensors located on the ER membrane: protein kinase RNA-like ER kinase (PERK), inositol requiring protein-1α (IRE1α) and activating transcription factor-6 (ATF6). When the unfolded/misfolded proteins in the ER accumulated, the ER chaperone BiP/GRP78 is dissociated from the luminal domains of the ER stress sensors to activate three transmembrane proteins. Each mechanism, upon activation, causes downstream reactions, such as the PERK-eIF2α-ATF4-CHOP signaling pathway, IRE1-TRAF2 signaling pathway, ASK1 signaling pathway and ATF6 signaling pathway (28). Thus triggering UPR, which includes slowing mRNA translation, increasing mRNA degradation, decreasing new protein synthesis, enhancing unfolded protein folding, and encouraging misfolded protein degradation to alleviate ER stress (29). Nonetheless, severe and chronic ER stress may result in aberrant activation of the UPR, which ultimately leads to apoptosis or autophagy-dependent cell death (30). In addition to UPR, ER over response (EOR) and sterol response element binding protein steroid regulatory cascade (SREBP) are essential components of ER stress, and there is a complex interaction between the three to reduce ER stress.

Under DN status, hyperglycemia, proteinuria, FFA, and AGEs disrupt proteostasis, resulting in the accumulation of unfolded/misfolded proteins in the ER lumen, thereby inducing excessive ER stress in renal intrinsic cells and promoting the activation and interaction of autophagy, apoptosis, inflammation, and oxidative stress-related pathways mediated by ER stress (31, 32). Which could have a crucial role in the etiology and progression of DN. ER stress is well-documented in DN, mRNAs encoding several ER chaperones were shown to be higher in the kidneys of humans with DN (33), for instance, recent studies shed light on the crosstalk between ER stress and oxidative stress in peripheral blood mononuclear cells (PBMC) of DN subjects, and significantly contributing to the onset and progression of DN (34). Importantly, the ER stress-mediated mechanism offers DN patients a possible treatment target. By inhibiting Fyn kinase-mediated ER stress, the Pan-Src kinase inhibitor described by Dorotea et al. (35) reduces proximal tubular cell damage in a diabetic milieu. In addition, Zhong et al. (36) discovered that dioscin protected against DN by decreasing oxidative stress, inflammation, and apoptosis caused by mitochondrial and ER stress. Nonetheless, the specific biological activities and immune-related molecular patterns of ER stress in DN are not entirely understood.

Using bioinformatics analysis, we built a comprehensive and in-depth evaluation system for ERSRGs and biochemical pathways involved in DN patients. Firstly, a total of 497 DEGs were detected between 90 DN patients and 100 healthy controls using the GEO database, revealing 255 upregulated genes and 242 downregulated genes. Subsequent GO enrichment analysis results supported that extracellular matrix (ECM) deposition and immune response may be involved in DN, whereas KEGG enrichment analysis demonstrated that oxidative stress and inflammatory reaction were closely related to the pathological changes of DN, which is consistent with previous research, similarly, DEGs were closely related to the pathological changes of DN. Next, we utilize the WGCNA to weight and classify co-expressed genes in multi-chip datasets, with thirteen modules listed. Each module and its associated traits are ultimately connected, so identifying module genes with the strongest relationships with DN samples for future examination. As a results, we found that 49 hub ERSRGs were strongly related with DN, the focus of our investigation, by comparing the ERSRGs in the database with those reported in the literature.

Additional enrichment analysis revealed the biological functions and pathways mediated by all hub ERSRGs, with the imbalance in ECM synthesis and degradation was particularly remarkable. The chronic infiltration of an immunological microinflammatory state in DN and the persistent stimulation of hyperglycemia prolong the repair of ECM protein following injury, resulting in pathological alterations. Renal fibrosis resulted from the excessive accumulation of ECM protein (37). Recent experimental evidence suggests that a megacluster of miRNAs (including miR-379 and others) and its host lncRNA (lncMGC) are increased by ER stress in the kidneys of diabetic mice and cause ECM accumulation of DN (38–40). In the second place, current evidence suggests that AGEs and ER stress are mutually induced in the pathophysiology of hyperglycemia, hypoxia, oxidative stress, RAGE-mediated inflammation, and aging in a variety of metabolic diseases (41). AGE exposure elevates the ER stress marker GRP78 and changes the ER protein folding sensor proteins, while targeting advanced glycation may be advantageous for ER homeostasis maintenance (42). In addition, the disruption of the insulin-PI3K-Akt signaling pathway in podocytes of the kidney leads to ER stress, podocyte apoptosis, and proteinuria in DN (43). Other inflammation-mediated pathways, including the IL-17 signaling pathway, the HIF-1 signaling pathway, and the Toll-like receptor signaling, were activated in response to ER stress (44), which may be associated with oxidative stress and chronic inflammation in renal tissue. It is concluded that ER stress is indisputable in the pathophysiology of DN.

Increasingly, machine learning algorithms are utilized to develop decision models that aid in the detection and treatment of disease. Five possible biomarkers linking DN and ER stress (CDKN1B, EGR1, FKBP5, GDF15, and MARCKS) were tested in the current study after merging the findings of three machine learning models and additional selection of verification sets. Moreover, although we confirmed that all five characteristic genes expression level can be used as an independent diagnostic marker, we intend to develop a more comprehensive diagnosis pattern by converting it into a score and taking all these five characteristic genes into consideration. Then, a nomogram was created and showed the improvement of diagnostic efficacy, but its external validity was poor in external validation, which may restrict its effective in clinical applications for DN diagnosis.

Cyclin-dependent kinase inhibitor 1B (CDKN1B), also known as p27^Kip1^, slows cell cycle transition following DN, causing cells to remain in the G1 phase and inhibiting cell proliferation (45). The CDKN1B upregulation has been linked to glomerular hypertrophy, mesangial expansion, and ECM deposition, whereas downregulation could slow the course of DN (46), according to studies. Dong et al. (47) revealed the decrease of CDKN1B mRNA expression in the glomeruli of DN patients in the Nephroseq data set and confirmed that the expression level of CDKN1B mRNA in podocytes decreases gradually as glucose concentration rises, which is consistent with our findings. Additionally, it has been established that CDKN1B upregulation can inhibit ER stress-induced apoptosis (48). All evidence suggests that CDKN1B may play a crucial role in the pathophysiology of DN. Early growth response-1 (EGR1) is an immediate-early transcription factor that has been demonstrated to contribute to diabetic atherosclerosis by boosting ECM synthesis through interaction with TGF-β and promoting proinflammatory responses (49). Fan et al. (50) observed a reduction in the expression of EGR1 mRNA in the DN. Cheong et al. (51) discovered a correlation between EGR1 expression and genes for ER stress and anti-apoptosis in human pancreatic tissues. However, more research has to be done on EGR1, a promising clinical indication of DN under ER stress. In reaction to stress, FK506-binding protein 51 (FKBP5) modifies the sensitivity of the glucocorticoid receptor. Additionally, the pathophysiology of DN has been connected to aberrant FKBP5 methylation. Lee et al. (52) discovered that the expression of FKBP5 mRNA was elevated in the urine of DN patients, which may account for the reduction of FKBP5 in renal tissue samples of DN observed in this investigation.

The TGF-β family member growth differentiation factor-15 (GDF15) is emerging as a diagnostic and therapeutic target for metabolic disorders (53). In preclinical kidney injury, kidney GDF15 expression appears to have a protective role, since GDF15-deficient diabetic animals exhibited more severe interstitial damage (54, 55). In humans with DN, the expression of GDF15 in plasma and urine has been discovered as a possible biomarker for early diagnosis of DN, and has been shown to independently correlate with renal risk in prior research (56–58). Meanwhile, the kidney was hypothesized to be a source of circulating and urine GDF15 (59). Also reported is the pathophysiological function of GDF15 in regulating ER stress. Through UPR signaling, ER stress promotes GDF15 expression and release (60). Moreover, ablation of GDF15 lowers ER stress-induced β-cell apoptosis in diabetes (61). These findings suggest that GDF15 is a possible diagnostic marker for DN and may play a crucial role in its progression. Myristoylated alanine-rich C kinase substrate (MARCKS) is a biological substrate with high affinity for protein kinase C (PKC), with one of its most essential functions being to provide PI3K with PIP2 pools and so activate AKT (62). To yet, however, no research on the role of MARCKS in DN have been documented. Hence, the association between them remains unknown. To sum up, based on these five characteristic genes closely related to the progression of DN, we may formulate a new diagnosis workup, estimate prognosis, and provide targeted treatment in the clinical, which could provide a new therapeutic target for DN and contribute to personalized medicine.

Despite the fact that DN is not a “immune-mediated” kidney disease, numerous studies have shown that both innate and adaptive immune pathways can promote or control renal function degradation in DN (63). We found significant differences in the type and abundance of infiltrating immune cell populations between the two groups, including B cells memory, T cells gamma delta, NK cells activated, Macrophages M2, Dendritic cells resting, and Mast cells resting, among others, highlighting the importance of immune cells in the development of DN. In the meantime, it was discovered that all five of these characteristic genes are implicated in immune cell infiltration during DN glomerular damage. Improving aberrant immunological status by focusing on them may be a promising therapy strategy for DN. In addition, we generated two subtypes based on the expression profiling of five distinctive ER stress regulators using an unsupervised cluster approach. Analysis of functional enrichment revealed that subtype2 was closely associated with TGF-β signaling pathway (64), WNT signaling pathway (65), and ECM deposition (66), which were shown to mediate excessive ER stress. Therefore, it is plausible to infer that subtype2 may be more closely associated with ER-stress, which could aid in the early detection and treatment of DN. Finally, CMap identified potential small-molecule medicines that could reverse the expression of ERSRGs. Mycophenolate-mofetil and honokiol have been demonstrated to slow the course of DN (67, 68), although the underlying mechanism remains unknown.

Our study has a number of limitations. First, the present study based on public open-source databases; additional clinical and experimental studies on the identification of ER stress-related biomarkers, as well as the scope and precision of particular applications, are required. In addition, several clinicopathological characteristics, such as particular clinical classification, follow-up information, and complications, are not taken into account in our research, necessitating additional clinical investigation. Last but not least, more research is needed to fully understand the possible effects because there hasn’t been much documented on the molecular interactions between these five characteristic genes and immune cells.

## 5 Conclusions

In summary, our study provides new insights into the role of ER stress in the pathophysiology of DN and the development of new targets for early diagnosis and treatment of DN. In addition, five characteristic genes (CDKN1B, EGR1, FKBP5, GDF15, and MARCKS) have been preliminarily identified as sensitive potential biomarkers that could influence the development of DN by controlling ER stress. Further research is required to determine the precise molecular mechanism and functional pathway of these proteins in DN.

## Data availability statement

The datasets presented in this study can be found in online repositories. The names of the repository/repositories and accession number(s) can be found in the article/**Supplementary Material**.

## Author contributions

JMS made a statistical analysis of the data and interpreted the results as a major contributor in writing the manuscript. JP, LW, HDX, YZ, and HMC finished the data acquisition and processing of the thesis together. YS, YG, YCZ, YXG, and ZXD completed data analysis and discussion. XHZ and HFL provided ideas for articles and guided writing. All authors contributed to the article and approved the submitted version.
