# Supplementary material for: Identification of endoplasmic reticulum stress-related biomarkers of diabetes nephropathy based on bioinformatics and machine learning
Source: Front Endocrinol (Lausanne). 2023 Sep 1;14:1206154. doi: 10.3389/fendo.2023.1206154 (PMC10513048; doi:10.3389/fendo.2023.1206154)
Supplement: Supplementary file 1 [file DataSheet_1.docx]

Supplementary Material

Identification of endoplasmic reticulum stress-related biomarkers of diabetes nephropathy based on bioinformatics and machine learning

Jiaming SU, Jing PENG, Lin WANG

*** Correspondence:** Hongfang Liu, E-mail: a2954@bucm.edu.cn.

# Supplementary Figures

**
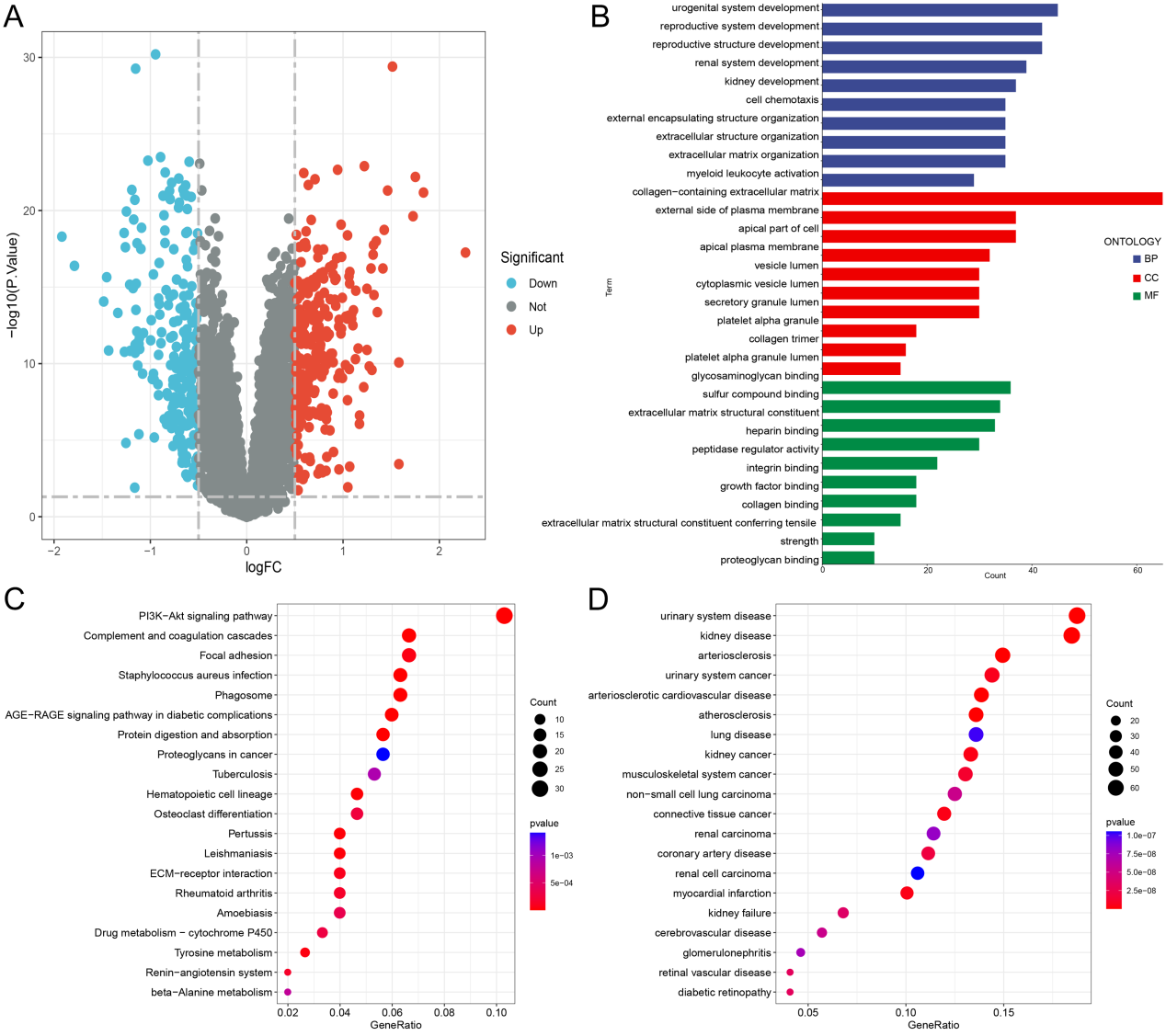
**

**Supplementary Figure 1.** Identification and functional enrichment analysis of DEGs. (**A**) Volcano plots of DEGs. (**B**) GO enrichment analysis of DEGs. (**C**) KEGG enrichment analysis of DEGs. (**D**) DO enrichment analysis of DEGs.


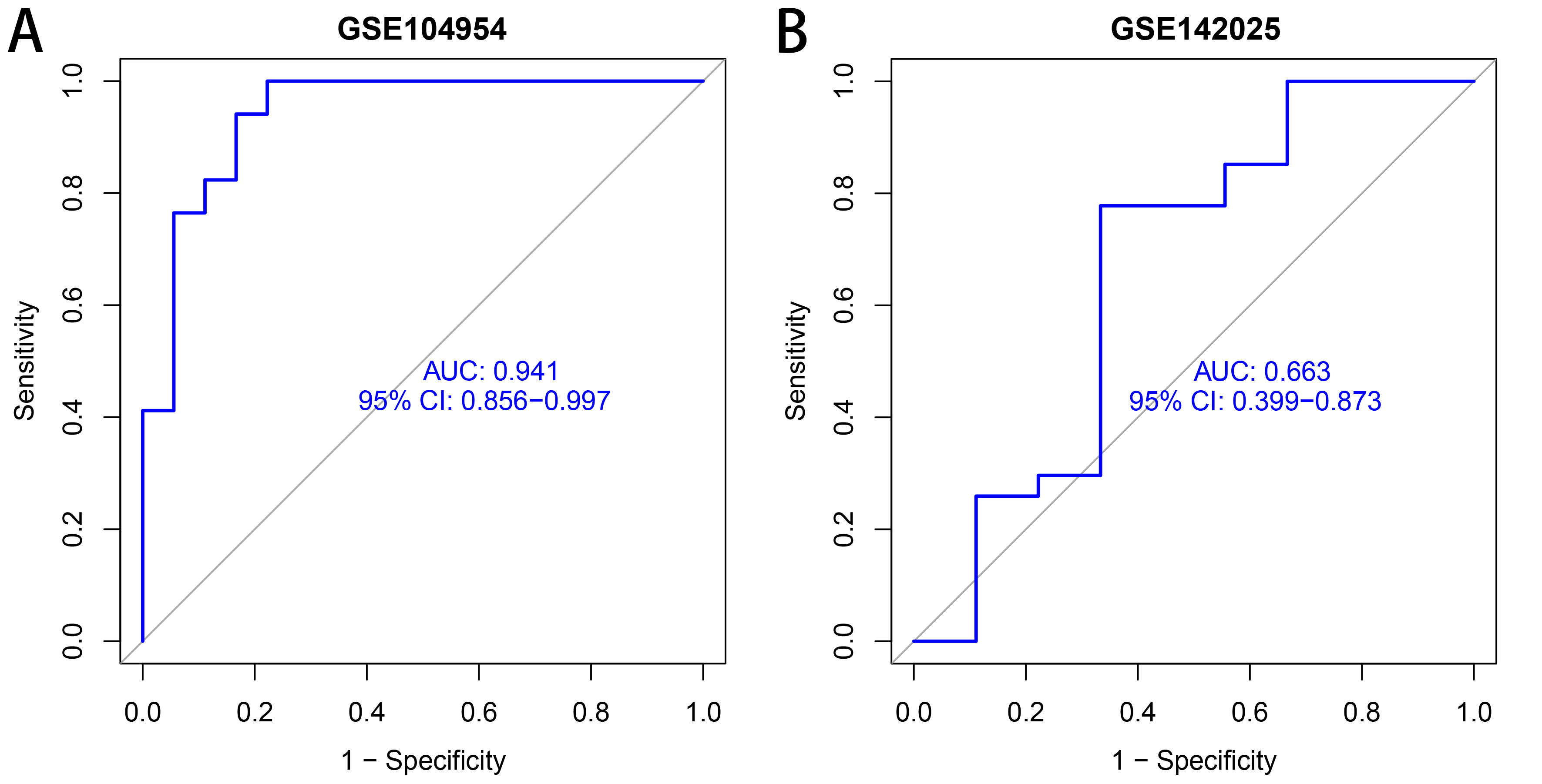


**Supplementary Figure 2.** External validation of the model of prediction nomogram. (**A**) The ROC curves for evaluating the diagnostic performance in validation dataset of tubulointerstitium (GSE104954). (**B**) The ROC curves for evaluating the diagnostic performance in validation dataset of kidney biopsy (GSE142025).

# Supplementary Tables

**Supplementary TABLE 1** The exact sample assignments of the multi-chip dataset and validation data set.

| Sample | set | condition |
| --- | --- | --- |
| GSM2810645 | training | diseased |
| GSM2810646 | training | diseased |
| GSM2810647 | training | diseased |
| GSM2810648 | training | diseased |
| GSM2810649 | training | diseased |
| GSM2810770 | training | diseased |
| GSM2810771 | training | diseased |
| GSM2810772 | training | diseased |
| GSM2810773 | training | diseased |
| GSM2810774 | training | diseased |
| GSM2810775 | training | diseased |
| GSM2810776 | training | diseased |
| GSM756992 | training | diseased |
| GSM756993 | training | diseased |
| GSM756994 | training | diseased |
| GSM756995 | training | diseased |
| GSM756996 | training | diseased |
| GSM756997 | training | diseased |
| GSM756998 | training | diseased |
| GSM756999 | training | diseased |
| GSM757000 | training | diseased |
| GSM1146269 | training | diseased |
| GSM1146270 | training | diseased |
| GSM1146271 | training | diseased |
| GSM1146272 | training | diseased |
| GSM1146273 | training | diseased |
| GSM1146274 | training | diseased |
| GSM1146275 | training | diseased |
| GSM1146201 | training | diseased |
| GSM1146202 | training | diseased |
| GSM1146203 | training | diseased |
| GSM1146204 | training | diseased |
| GSM1146205 | training | diseased |
| GSM1146206 | training | diseased |
| GSM1146207 | training | diseased |
| GSM2544275 | training | diseased |
| GSM2544276 | training | diseased |
| GSM2544277 | training | diseased |
| GSM2544278 | training | diseased |
| GSM2544279 | training | diseased |
| GSM2544280 | training | diseased |
| GSM2544281 | training | diseased |
| GSM2544282 | training | diseased |
| GSM2544283 | training | diseased |
| GSM2544284 | training | diseased |
| GSM2544285 | training | diseased |
| GSM2544286 | training | diseased |
| GSM2544287 | training | diseased |
| GSM2544288 | training | diseased |
| GSM2544289 | training | diseased |
| GSM2544290 | training | diseased |
| GSM2544291 | training | diseased |
| GSM2544292 | training | diseased |
| GSM2544293 | training | diseased |
| GSM2544294 | training | diseased |
| GSM2544295 | training | diseased |
| GSM2544296 | training | diseased |
| GSM2544297 | training | diseased |
| GSM2544298 | training | diseased |
| GSM2544299 | training | diseased |
| GSM2544300 | training | diseased |
| GSM2544301 | training | diseased |
| GSM2544302 | training | diseased |
| GSM2544303 | training | diseased |
| GSM2544304 | training | diseased |
| GSM2544305 | training | diseased |
| GSM2544306 | training | diseased |
| GSM2544307 | training | diseased |
| GSM2544308 | training | diseased |
| GSM2544309 | training | diseased |
| GSM2544310 | training | diseased |
| GSM2544311 | training | diseased |
| GSM2544312 | training | diseased |
| GSM2544313 | training | diseased |
| GSM2544314 | training | diseased |
| GSM2544315 | training | diseased |
| GSM2642291 | training | diseased |
| GSM2642292 | training | diseased |
| GSM2642293 | training | diseased |
| GSM2642294 | training | diseased |
| GSM2642295 | training | diseased |
| GSM2642296 | training | diseased |
| GSM2642297 | training | diseased |
| GSM2642424 | training | diseased |
| GSM2642425 | training | diseased |
| GSM2642426 | training | diseased |
| GSM2642427 | training | diseased |
| GSM2642428 | training | diseased |
| GSM2642429 | training | diseased |
| GSM2642430 | training | diseased |
| GSM2810677 | training | healthy |
| GSM2810678 | training | healthy |
| GSM2810714 | training | healthy |
| GSM2810715 | training | healthy |
| GSM2810716 | training | healthy |
| GSM2810793 | training | healthy |
| GSM2810794 | training | healthy |
| GSM2810795 | training | healthy |
| GSM2810796 | training | healthy |
| GSM2810797 | training | healthy |
| GSM2810798 | training | healthy |
| GSM2810799 | training | healthy |
| GSM2810800 | training | healthy |
| GSM2810801 | training | healthy |
| GSM2810802 | training | healthy |
| GSM2810803 | training | healthy |
| GSM2810804 | training | healthy |
| GSM2810805 | training | healthy |
| GSM2810806 | training | healthy |
| GSM2810807 | training | healthy |
| GSM2810808 | training | healthy |
| GSM2810809 | training | healthy |
| GSM2810810 | training | healthy |
| GSM2810838 | training | healthy |
| GSM2810839 | training | healthy |
| GSM2810840 | training | healthy |
| GSM758485 | training | healthy |
| GSM758486 | training | healthy |
| GSM758487 | training | healthy |
| GSM758488 | training | healthy |
| GSM758489 | training | healthy |
| GSM758490 | training | healthy |
| GSM758491 | training | healthy |
| GSM758492 | training | healthy |
| GSM758493 | training | healthy |
| GSM758494 | training | healthy |
| GSM758495 | training | healthy |
| GSM758496 | training | healthy |
| GSM758497 | training | healthy |
| GSM757001 | training | healthy |
| GSM757002 | training | healthy |
| GSM757003 | training | healthy |
| GSM757004 | training | healthy |
| GSM757005 | training | healthy |
| GSM757006 | training | healthy |
| GSM757007 | training | healthy |
| GSM757008 | training | healthy |
| GSM757009 | training | healthy |
| GSM757010 | training | healthy |
| GSM757011 | training | healthy |
| GSM757012 | training | healthy |
| GSM757013 | training | healthy |
| GSM1146320 | training | healthy |
| GSM1146321 | training | healthy |
| GSM1146322 | training | healthy |
| GSM1146215 | training | healthy |
| GSM1146216 | training | healthy |
| GSM1146217 | training | healthy |
| GSM1146218 | training | healthy |
| GSM1146219 | training | healthy |
| GSM1146220 | training | healthy |
| GSM1146221 | training | healthy |
| GSM1146222 | training | healthy |
| GSM1146229 | training | healthy |
| GSM1146230 | training | healthy |
| GSM1146231 | training | healthy |
| GSM1146232 | training | healthy |
| GSM1146233 | training | healthy |
| GSM1146234 | training | healthy |
| GSM2544316 | training | healthy |
| GSM2544317 | training | healthy |
| GSM2544318 | training | healthy |
| GSM2544319 | training | healthy |
| GSM2544320 | training | healthy |
| GSM2544321 | training | healthy |
| GSM2544322 | training | healthy |
| GSM2544323 | training | healthy |
| GSM2544324 | training | healthy |
| GSM2544325 | training | healthy |
| GSM2544326 | training | healthy |
| GSM2544327 | training | healthy |
| GSM2544328 | training | healthy |
| GSM2544329 | training | healthy |
| GSM2544330 | training | healthy |
| GSM2544331 | training | healthy |
| GSM2544332 | training | healthy |
| GSM2544333 | training | healthy |
| GSM2544334 | training | healthy |
| GSM2544335 | training | healthy |
| GSM2642475 | training | healthy |
| GSM2642476 | training | healthy |
| GSM2642477 | training | healthy |
| GSM2642305 | training | healthy |
| GSM2642306 | training | healthy |
| GSM2642307 | training | healthy |
| GSM2642330 | training | healthy |
| GSM2642331 | training | healthy |
| GSM2642332 | training | healthy |
| GSM2642333 | training | healthy |
| GSM2642334 | training | healthy |
| GSM2811029 | validation | diseased |
| GSM2811030 | validation | diseased |
| GSM2811031 | validation | diseased |
| GSM2811032 | validation | diseased |
| GSM2811033 | validation | diseased |
| GSM2811034 | validation | diseased |
| GSM2811035 | validation | diseased |
| GSM2810894 | validation | diseased |
| GSM2810895 | validation | diseased |
| GSM2810896 | validation | diseased |
| GSM2810897 | validation | diseased |
| GSM2810898 | validation | diseased |
| GSM2810899 | validation | diseased |
| GSM2810900 | validation | diseased |
| GSM2810901 | validation | diseased |
| GSM2810902 | validation | diseased |
| GSM2810903 | validation | diseased |
| GSM2811043 | validation | healthy |
| GSM2811044 | validation | healthy |
| GSM2811045 | validation | healthy |
| GSM2811046 | validation | healthy |
| GSM2811047 | validation | healthy |
| GSM2811048 | validation | healthy |
| GSM2811049 | validation | healthy |
| GSM2811050 | validation | healthy |
| GSM2811051 | validation | healthy |
| GSM2811052 | validation | healthy |
| GSM2811053 | validation | healthy |
| GSM2811054 | validation | healthy |
| GSM2811055 | validation | healthy |
| GSM2811056 | validation | healthy |
| GSM2811057 | validation | healthy |
| GSM2811058 | validation | healthy |
| GSM2811059 | validation | healthy |
| GSM2811060 | validation | healthy |
| GSM4217781 | validation | diseased |
| GSM4217782 | validation | diseased |
| GSM4217783 | validation | diseased |
| GSM4217784 | validation | diseased |
| GSM4217785 | validation | diseased |
| GSM4217786 | validation | diseased |
| GSM4217787 | validation | diseased |
| GSM4217788 | validation | diseased |
| GSM4217789 | validation | diseased |
| GSM4217790 | validation | diseased |
| GSM4217791 | validation | diseased |
| GSM4217792 | validation | diseased |
| GSM4217793 | validation | diseased |
| GSM4217794 | validation | diseased |
| GSM4217795 | validation | diseased |
| GSM4217796 | validation | diseased |
| GSM4217797 | validation | diseased |
| GSM4217798 | validation | diseased |
| GSM4217799 | validation | diseased |
| GSM4217800 | validation | diseased |
| GSM4217801 | validation | diseased |
| GSM4217802 | validation | diseased |
| GSM4217803 | validation | diseased |
| GSM4217804 | validation | diseased |
| GSM4217805 | validation | diseased |
| GSM4217806 | validation | diseased |
| GSM4217807 | validation | diseased |
| GSM4217808 | validation | healthy |
| GSM4217809 | validation | healthy |
| GSM4217810 | validation | healthy |
| GSM4217811 | validation | healthy |
| GSM4217812 | validation | healthy |
| GSM4217813 | validation | healthy |
| GSM4217814 | validation | healthy |
| GSM4217815 | validation | healthy |
| GSM4217816 | validation | healthy |

**Supplementary TABLE 2** 1406 ER stress-related genes (ERSRGs) obtained from MSigDB and GeneCards database.

| Symbol | Description |
| --- | --- |
| ABCA1 | ATP binding cassette subfamily A member 1 |
| ABCA7 | ATP binding cassette subfamily A member 7 |
| ABCC6 | ATP binding cassette subfamily C member 6 |
| ABCC8 | ATP binding cassette subfamily C member 8 |
| ABCD1 | ATP binding cassette subfamily D member 1 |
| ABCD4 | ATP binding cassette subfamily D member 4 |
| ABCG1 | ATP binding cassette subfamily G member 1 |
| ABL1 | ABL proto-oncogene 1, non-receptor tyrosine kinase |
| ACBD3 | Acyl-Coenzyme A binding domain containing 3, isoform CRA_a |
| ACE | Angiotensin converting enzyme |
| ACER1 | Alkaline ceramidase 1 (AlkCDase 1) (Alkaline CDase 1) (EC 3.5.1.-) (EC 3.5.1.23) (Acylsphingosine deacylase 3) (N-acylsphingosine amidohydrolase 3) |
| ACER3 | Alkaline ceramidase (EC 3.5.1.-) |
| ACP1 | Low molecular weight phosphotyrosine protein phosphatase (LMW-PTP) (LMW-PTPase) (EC 3.1.3.48) (Adipocyte acid phosphatase) (Low molecular weight cytosolic acid phosphatase) (EC 3.1.3.2) (Red cell acid phosphatase 1) |
| ACSF3 | Acyl-CoA synthetase family member 3 |
| ACSL3 | Acyl-CoA synthetase long chain family member 3 |
| ACSL4 | Acyl-CoA synthetase long chain family member 4 |
| ACTA1 | Actin alpha 1 skeletal muscle protein |
| ACTB | Actin, cytoplasmic 1 |
| ACTC1 | Actin alpha cardiac muscle 1 |
| ACTG1 | Actin gamma 1 |
| ADAMTS13 | ADAM metallopeptidase with thrombospondin type 1 motif 13 |
| ADAMTSL1 | ADAMTS-like protein 1 (ADAMTSL-1) (Punctin-1) |
| ADCYAP1 | Pituitary adenylyl cyclase activating protein |
| ADCYAP1R1 | Pituitary adenylate cyclase-activating polypeptide type I receptor (PACAP type I receptor) (PACAP-R-1) (PACAP-R1) |
| ADIPOQ | Adiponectin (30 kDa adipocyte complement-related protein) (Adipocyte complement-related 30 kDa protein) (ACRP30) (Adipocyte, C1q and collagen domain-containing protein) (Adipose most abundant gene transcript 1 protein) (apM-1) (Gelatin-binding protein) |
| ADRB2 | Beta-2 adrenergic receptor (Beta-2 adrenoceptor) (Beta-2 adrenoreceptor) |
| AFG3L2 | AFG3 like matrix AAA peptidase subunit 2 |
| AGER | Advanced glycosylation end product-specific receptor |
| AGPAT1 | 1-acyl-sn-glycerol-3-phosphate acyltransferase alpha (EC 2.3.1.51) (1-acylglycerol-3-phosphate O-acyltransferase 1) (1-AGP acyltransferase 1) (1-AGPAT 1) (Lysophosphatidic acid acyltransferase alpha) (LPAAT-alpha) (Protein G15) |
| AGPAT2 | 1-acyl-sn-glycerol-3-phosphate acyltransferase beta (EC 2.3.1.51) (1-acylglycerol-3-phosphate O-acyltransferase 2) (1-AGP acyltransferase 2) (1-AGPAT 2) (Lysophosphatidic acid acyltransferase beta) (LPAAT-beta) |
| AGR2 | Anterior gradient 2, protein disulphide isomerase family member |
| AGR3 | Anterior gradient 3, protein disulphide isomerase family member |
| AGRN | Agrin |
| AGT | Truncated angiotensinogen |
| AGTR1 | Type-1 angiotensin II receptor |
| AHCYL1 | Adenosylhomocysteinase (EC 3.13.2.1) |
| AIFM1 | Apoptosis inducing factor mitochondria associated 1 |
| AIMP1 | Aminoacyl tRNA synthetase complex interacting multifunctional protein 1 |
| AKAP6 | A-kinase anchoring protein 6 |
| AKAP9 | A-kinase anchoring protein 9 |
| AKR1B1 | Aldo-keto reductase family 1 member B |
| AKT1 | AKT serine/threonine kinase 1 |
| ALB | Albumin |
| ALDH3A2 | Aldehyde dehydrogenase 3 family member A2 |
| ALG1 | ALG1 chitobiosyldiphosphodolichol beta-mannosyltransferase |
| ALG11 | ALG11 alpha-1,2-mannosyltransferase |
| ALG13 | ALG13 UDP-N-acetylglucosaminyltransferase subunit |
| ALG14 | UDP-N-acetylglucosamine transferase subunit ALG14 homolog |
| ALG2 | ALG2 alpha-1,3/1,6-mannosyltransferase |
| ALOX15 | Polyunsaturated fatty acid lipoxygenase ALOX15 (12/15-lipoxygenase) (Arachidonate 12-lipoxygenase, leukocyte-type) (12-LOX) (EC 1.13.11.31) (Arachidonate 15-lipoxygenase) (15-LOX) (15-LOX-1) (EC 1.13.11.33) (Arachidonate omega-6 lipoxygenase) (Hepoxilin A3 synthase Alox15) (EC 1.13.11.-) (Linoleate 13S-lipoxygenase) (EC 1.13.11.12) |
| ALOX5 | Polyunsaturated fatty acid 5-lipoxygenase |
| ALPL | Alkaline phosphatase, tissue-nonspecific isozyme (AP-TNAP) (TNS-ALP) (TNSALP) (EC 3.1.3.1) (Alkaline phosphatase liver/bone/kidney isozyme) (Phosphoamidase) (Phosphocreatine phosphatase) (EC 3.9.1.1) |
| ALPP | Alkaline phosphatase, placental type (EC 3.1.3.1) (Alkaline phosphatase Regan isozyme) (Placental alkaline phosphatase 1) (PLAP-1) |
| AMFR | Autocrine motility factor receptor |
| ANK1 | Ankyrin 1 |
| ANK2 | Ankyrin 2 |
| ANKLE2 | Ankyrin repeat and LEM domain containing 2 |
| ANKS4B | Ankyrin repeat and SAM domain-containing protein 4B (Harmonin-interacting ankyrin repeat-containing protein) (Harp) |
| ANKZF1 | Ankyrin repeat and zinc finger peptidyl tRNA hydrolase 1 |
| ANXA2 | Annexin A2 |
| ANXA5 | Annexin A5 |
| APAF1 | Apoptotic protease-activating factor 1 (APAF-1) |
| APEX1 | Apurinic/apyrimidinic endodeoxyribonuclease 1 |
| APOA1 | Apolipoprotein A-I (Apo-AI) (ApoA-I) (Apolipoprotein A1) [Cleaved into: Proapolipoprotein A-I (ProapoA-I); Truncated apolipoprotein A-I (Apolipoprotein A-I(1-242))] |
| APOB | Apolipoprotein B |
| APOE | Apolipoprotein E (Apo-E) |
| APP | Amyloid-beta A4 protein |
| AQP11 | Aquaporin-11 (AQP-11) |
| ARF1 | ADP ribosylation factor 1 |
| ARFGAP2 | ADP ribosylation factor GTPase activating protein 2 |
| ARL6IP1 | ADP ribosylation factor like GTPase 6 interacting protein 1 |
| ARL6IP5 | PRA1 family protein |
| ARRB1 | Arrestin beta 1 |
| ARSA | Arylsulfatase A |
| ARSH | Arylsulfatase H (ASH) (EC 3.1.6.-) |
| ASL | Argininosuccinate lyase |
| ASPH | Aspartate beta-hydroxylase |
| ATF1 | Cyclic AMP-dependent transcription factor ATF-1 (cAMP-dependent transcription factor ATF-1) (Activating transcription factor 1) (Protein TREB36) |
| ATF2 | Cyclic AMP-dependent transcription factor ATF-2 (cAMP-dependent transcription factor ATF-2) (Activating transcription factor 2) (Cyclic AMP-responsive element-binding protein 2) (CREB-2) (cAMP-responsive element-binding protein 2) (HB16) (cAMP response element-binding protein CRE-BP1) |
| ATF3 | Cyclic AMP-dependent transcription factor ATF-3 (cAMP-dependent transcription factor ATF-3) (Activating transcription factor 3) |
| ATF4 | Cyclic AMP-dependent transcription factor ATF-4 (cAMP-dependent transcription factor ATF-4) (Activating transcription factor 4) (Cyclic AMP-responsive element-binding protein 2) (CREB-2) (cAMP-responsive element-binding protein 2) (Tax-responsive enhancer element-binding protein 67) (TaxREB67) |
| ATF6 | Cyclic AMP-dependent transcription factor ATF-6 alpha (cAMP-dependent transcription factor ATF-6 alpha) (Activating transcription factor 6 alpha) (ATF6-alpha) [Cleaved into: Processed cyclic AMP-dependent transcription factor ATF-6 alpha] |
| ATF6B | Cyclic AMP-dependent transcription factor ATF-6 beta |
| ATG10 | Autophagy related 10 |
| ATG14 | Beclin 1-associated autophagy-related key regulator (Barkor) (Autophagy-related protein 14-like protein) (Atg14L) |
| ATG7 | Ubiquitin-like modifier-activating enzyme ATG7 (ATG12-activating enzyme E1 ATG7) (Autophagy-related protein 7) (APG7-like) (hAGP7) (Ubiquitin-activating enzyme E1-like protein) |
| ATG9A | Autophagy-related protein 9 |
| ATL1 | Atlastin GTPase 1 |
| ATL2 | Atlastin GTPase 2 |
| ATL3 | Atlastin GTPase 3 |
| ATM | Serine-protein kinase ATM (EC 2.7.11.1) (Ataxia telangiectasia mutated) (A-T mutated) |
| ATP13A1 | ATPase 13A1 |
| ATP13A2 | ATP13A2 protein |
| ATP1A1 | ATPase Na+/K+ transporting subunit alpha 1 |
| ATP1A3 | ATPase Na+/K+ transporting subunit alpha 3 |
| ATP2A1 | ATPase sarcoplasmic/endoplasmic reticulum Ca2+ transporting 1 |
| ATP2A2 | ATPase sarcoplasmic/endoplasmic reticulum Ca2+ transporting 2 |
| ATP2A3 | ATPase sarcoplasmic/endoplasmic reticulum Ca2+ transporting 3 |
| ATP2C1 | ATPase secretory pathway Ca2+ transporting 1 |
| ATP5MK | ATP synthase membrane subunit K, mitochondrial (ATP synthase membrane subunit DAPIT, mitochondrial) (Diabetes-associated protein in insulin-sensitive tissues) (HCV F-transactivated protein 2) (Up-regulated during skeletal muscle growth protein 5) |
| ATP7A | ATPase copper transporting alpha |
| ATR | ATR serine/threonine kinase |
| ATXN2 | Ataxin 2 |
| ATXN2L | Ataxin 2 like |
| ATXN3 | Ataxin 3 |
| AUP1 | Lipid droplet-regulating VLDL assembly factor AUP1 (Ancient ubiquitous protein 1) |
| AVP | Arginine vasopressin |
| B2M | Beta-2-microglobulin |
| BACE1 | Beta-secretase 1 (EC 3.4.23.46) (Beta-site amyloid precursor protein cleaving enzyme 1) (Memapsin-2) (Membrane-associated aspartic protease 2) |
| BAD | BCL2 associated agonist of cell death |
| BAG3 | BAG cochaperone 3 |
| BAG6 | BAG cochaperone 6 |
| BAK1 | BCL2-antagonist/killer 1 isoform 2 |
| BAX | BCL2-associated X protein transcript variant delta2 |
| BBC3 | Bcl-2-binding component 3, isoforms 3/4 (JFY-1) (p53 up-regulated modulator of apoptosis) |
| BCAP29 | B-cell receptor-associated protein (BCR-associated protein) |
| BCAP31 | B-cell receptor-associated protein (BCR-associated protein) |
| BCHE | Carboxylic ester hydrolase (EC 3.1.1.-) |
| BCL2 | Apoptosis regulator Bcl-2 |
| BCL2L1 | Bcl-2-like protein 1 (Apoptosis regulator Bcl-X) |
| BCL2L10 | BCL2 like 10 |
| BCL2L11 | BCL2 like 11 |
| BDNF | Brain derived neurotrophic factor |
| BECN1 | Beclin 1 |
| BET1 | Bet1 golgi vesicular membrane trafficking protein |
| BFAR | Bifunctional apoptosis regulator |
| BGLAP | Osteocalcin (Bone Gla protein) (BGP) (Gamma-carboxyglutamic acid-containing protein) |
| BHLHA15 | Class A basic helix-loop-helix protein 15 (bHLHa15) (Class B basic helix-loop-helix protein 8) (bHLHb8) (Muscle, intestine and stomach expression 1) (MIST-1) |
| BID | BH3 interacting domain death agonist Si6 isoform |
| BIRC2 | Baculoviral IAP repeat containing 2 |
| BLZF1 | Basic leucine zipper nuclear factor 1 |
| BMP2 | Bone morphogenetic protein 2 |
| BNIP1 | BCL2 interacting protein 1 |
| BOK | Uncharacterized protein BOK |
| BRAF | B-Raf proto-oncogene, serine/threonine kinase |
| BRCA1 | Truncated breast and ovarian cancer susceptibility protein 1 |
| BRCA2 | Truncated breast and ovarian cancer susceptibility protein 2 |
| BRSK2 | BR serine/threonine kinase 2 |
| BSCL2 | Truncated seipin |
| BSG | Basigin (Ok blood group) |
| BTRC | Beta-transducin repeat containing isoform 1 |
| C1R | Complement C1r |
| C1S | Complement C1s |
| C2CD2L | C2CD2 like |
| C3orf52 | Chromosome 3 open reading frame 52 |
| C6orf120 | UPF0669 protein C6orf120 |
| C9orf72 | C9orf72-SMCR8 complex subunit |
| CACNA1A | Voltage-dependent P/Q-type calcium channel subunit alpha |
| CACNA1C | Voltage-dependent L-type calcium channel subunit alpha |
| CACNA1S | Voltage-dependent L-type calcium channel subunit alpha |
| CALHM1 | Calcium homeostasis modulator protein 1 (Protein FAM26C) |
| CALM1 | Calmodulin 1 |
| CALM2 | Calmodulin 2 |
| CALM3 | Calmodulin 3 |
| CALR | Calreticulin |
| CALR3 | Calreticulin 3 |
| CALU | Calumenin |
| CAMK2A | Calcium/calmodulin dependent protein kinase II alpha |
| CAMK2D | Calcium/calmodulin dependent protein kinase II delta |
| CAMK2G | Calcium/calmodulin dependent protein kinase II gamma |
| CAMLG | Calcium modulating ligand |
| CANT1 | Calcium activated nucleotidase 1 |
| CANX | Calnexin |
| CAPN2 | Calpain 2 |
| CAPN3 | Calpain 3 |
| CARD14 | Caspase recruitment domain family member 14 |
| CASC3 | CASC3 exon junction complex subunit |
| CASP1 | Caspase 1 |
| CASP12 | Inactive caspase-12 (CASP-12) |
| CASP2 | Caspase 2 |
| CASP3 | Caspase 3 |
| CASP4 | Caspase 4 |
| CASP7 | Caspase 7 |
| CASP8 | Caspase 8 |
| CASP9 | Caspase 9 |
| CASQ1 | Calsequestrin |
| CASQ2 | Calsequestrin-2 (Calsequestrin, cardiac muscle isoform) |
| CASR | Calcium sensing receptor |
| CAST | Calpastatin (Calpain inhibitor) |
| CAT | Catalase |
| CAV1 | Caveolin 1 |
| CAV3 | Caveolin-3 (M-caveolin) |
| CBY1 | Protein chibby homolog 1 |
| CCDC47 | Coiled-coil domain containing 47 |
| CCDC88A | Coiled-coil domain containing 88A |
| CCDC88B | Coiled-coil domain containing 88B |
| CCK | Cholecystokinin (CCK) [Cleaved into: Cholecystokinin-58 (CCK58); Cholecystokinin-58 desnonopeptide ((1-49)-CCK58); Cholecystokinin-39 (CCK39); Cholecystokinin-33 (CCK33); Cholecystokinin-25 (CCK25); Cholecystokinin-18 (CCK18); Cholecystokinin-12 (CCK12); Cholecystokinin-8 (CCK8); Cholecystokinin-7 (CCK7); Cholecystokinin-5 (CCK5)] |
| CCL2 | C-C motif chemokine 2 (HC11) (Monocyte chemoattractant protein 1) (Monocyte chemotactic and activating factor) (MCAF) (Monocyte chemotactic protein 1) (MCP-1) (Monocyte secretory protein JE) (Small-inducible cytokine A2) |
| CCL4 | C-C motif chemokine 4 (C-C motif chemokine ligand 4) (CC chemokine ligand 4d2) (Monocyte adherence-induced protein 5 alpha) |
| CCN2 | CCN family member 2 (Cellular communication network factor 2) (Connective tissue growth factor) (Hypertrophic chondrocyte-specific protein 24) (Insulin-like growth factor-binding protein 8) (IBP-8) (IGF-binding protein 8) (IGFBP-8) |
| CCND1 | Cyclin D1 |
| CD36 | Mutant thrombospondin receptor |
| CD4 | CD4 protein (CD4 receptor) |
| CD59 | CD59 blood group antigen variant |
| CD74 | CD74 molecule |
| CDC42 | Cell division cycle 42 |
| CDH1 | E-cadherin 1 |
| CDH2 | Cadherin 2 |
| CDIPT | CDP-diacylglycerol--inositol 3-phosphatidyltransferase |
| CDK1 | Cyclin dependent kinase 1 |
| CDK2 | Cyclin dependent kinase 2 |
| CDK5 | Cyclin-dependent kinase 5 isoform 2 |
| CDK5RAP3 | CDK5 regulatory subunit associated protein 3 |
| CDKAL1 | Threonylcarbamoyladenosine tRNA methylthiotransferase (EC 2.8.4.5) (CDK5 regulatory subunit-associated protein 1-like 1) (tRNA-t(6)A37 methylthiotransferase) |
| CDKN1A | Cyclin-dependent kinase inhibitor 1 (CDK-interacting protein 1) (Melanoma differentiation-associated protein 6) (MDA-6) (p21) |
| CDKN1B | Cyclin dependent kinase inhibitor 1B |
| CDKN2A | Cyclin-dependent kinase inhibitor 2A |
| CDKN3 | Cyclin-dependent kinase inhibitor 3 splice variant |
| CEBPB | Transcription factor C/EBP beta |
| CERS6 | Ceramide synthase 6 (CerS6) (LAG1 longevity assurance homolog 6) (Sphingoid base N-palmitoyltransferase CERS6) (EC 2.3.1.291) |
| CERT1 | Ceramide transporter 1 |
| CES1 | Liver carboxylesterase 1 |
| CFLAR | CASP8 and FADD like apoptosis regulator |
| CFTR | Cystic fibrosis transmembrane conductance regulator |
| CGRRF1 | Cell growth regulator with ring finger domain 1 |
| CHAC1 | ChaC glutathione specific gamma-glutamylcyclotransferase 1 |
| CHAT | Choline O-acetyltransferase |
| CHEK1 | Checkpoint kinase 1 |
| CHERP | Calcium homeostasis endoplasmic reticulum protein |
| CHP1 | Calcineurin like EF-hand protein 1 |
| CHRM3 | Cholinergic receptor muscarinic 3 |
| CHRNE | Cholinergic receptor nicotinic epsilon subunit |
| CIRBP | Cold inducible RNA binding protein |
| CISD2 | CDGSH iron-sulfur domain-containing protein 2 |
| CKAP4 | Cytoskeleton associated protein 4 |
| CLCC1 | Chloride channel CLIC-like protein 1 |
| CLCN1 | Chloride voltage-gated channel 1 |
| CLGN | Alternative protein CLGN |
| CLN3 | Mutant CLN3 |
| CLN5 | CLN5 intracellular trafficking protein |
| CLN6 | CLN6 transmembrane ER protein (Ceroid-lipofuscinosis neuronal 6 late infantile variant isoform 2) |
| CLN8 | CLN8 transmembrane ER and ERGIC protein |
| CLU | Clusterin |
| CNIH4 | Cornichon family AMPA receptor auxiliary protein 4 |
| CNR1 | Cannabinoid receptor 1 |
| COL13A1 | Collagen alpha-1(XIII) chain (COLXIIIA1) |
| COL1A1 | Collagen alpha-1(I) chain (Alpha-1 type I collagen) |
| COL2A1 | Collagen alpha-1(II) chain (Alpha-1 type II collagen) [Cleaved into: Collagen alpha-1(II) chain; Chondrocalcin] |
| COL4A1 | Collagen alpha-1(IV) chain [Cleaved into: Arresten] |
| COL7A1 | Collagen alpha-1(VII) chain (Long-chain collagen) (LC collagen) |
| COLGALT1 | Collagen beta(1-O)galactosyltransferase 1 |
| COMP | Cartilage oligomeric matrix protein (COMP) (Thrombospondin-5) (TSP5) |
| COMT | Catechol-O-methyltransferase |
| COPA | COPI coat complex subunit alpha |
| COPB1 | COPI coat complex subunit beta 1 |
| COPB2 | COPI coat complex subunit beta 2 |
| COPE | Coatomer subunit epsilon (Epsilon-coat protein) |
| COPG1 | COPI coat complex subunit gamma 1 |
| COPS5 | COP9 signalosome subunit 5 |
| CP | Ceruloplasmin |
| CPQ | Carboxypeptidase Q |
| CPT2 | Carnitine palmitoyltransferase 2 |
| CR1 | Complement receptor type 1 |
| CRAT | Carnitine O-acetyltransferase |
| CREB1 | cAMP responsive element binding protein 1 |
| CREB3 | Cyclic AMP-responsive element-binding protein 3 (CREB-3) (cAMP-responsive element-binding protein 3) (Leucine zipper protein) (Luman) (Transcription factor LZIP-alpha) [Cleaved into: Processed cyclic AMP-responsive element-binding protein 3 (N-terminal Luman) (Transcriptionally active form)] |
| CREB3L1 | cAMP responsive element binding protein 3 like 1 |
| CREB3L2 | cAMP responsive element binding protein 3 like 2 |
| CREB3L3 | Cyclic AMP-responsive element-binding protein 3-like protein 3 (cAMP-responsive element-binding protein 3-like protein 3) (Transcription factor CREB-H) [Cleaved into: Processed cyclic AMP-responsive element-binding protein 3-like protein 3] |
| CREB3L4 | Cyclic AMP-responsive element-binding protein 3-like protein 4 (cAMP-responsive element-binding protein 3-like protein 4) (Androgen-induced basic leucine zipper protein) (AIbZIP) (Attaching to CRE-like 1) (ATCE1) (Cyclic AMP-responsive element-binding protein 4) (CREB-4) (cAMP-responsive element-binding protein 4) (Transcript induced in spermiogenesis protein 40) (Tisp40) (hJAL) [Cleaved into: Processed cyclic AMP-responsive element-binding protein 3-like protein 4] |
| CREBBP | CREB binding protein |
| CREBRF | CREB3 regulatory factor |
| CREBZF | CREB/ATF bZIP transcription factor |
| CRH | Corticotropin releasing hormone isoform 2 |
| CRHR1 | Corticotropin-releasing factor receptor 1 |
| CRHR2 | Corticotropin releasing hormone receptor 2 |
| CRP | C-reactive protein |
| CRYAB | Crystallin alpha B |
| CSNK2A1 | Casein kinase II subunit alpha (CK II alpha) (EC 2.7.11.1) |
| CSTB | Cystatin B |
| CTH | Cystathionine gamma-lyase (CGL) (CSE) (EC 4.4.1.1) (Cysteine desulfhydrase) (Cysteine-protein sulfhydrase) (Gamma-cystathionase) (Homocysteine desulfhydrase) (EC 4.4.1.2) |
| CTNNB1 | Catenin beta 1 |
| CTSB | Cathepsin B |
| CTSD | Cathepsin D isoform 2 |
| CUL3 | Uncharacterized protein CUL3 |
| CXCL8 | C-X-C motif chemokine ligand 8 |
| CYB5A | Cytochrome b5 type A |
| CYB5R3 | Cytochrome b5 reductase 3 |
| CYB5R4 | Cytochrome b5 reductase 4 |
| CYBA | Cytochrome b-245 alpha chain |
| CYBB | Cytochrome b-245 beta polypeptide isoform 1 |
| CYCS | Cytochrome c |
| CYP17A1 | Cytochrome P450 17A1 |
| CYP19A1 | Cytochrome P450 family 19 subfamily A member 1 |
| CYP1A1 | Cytochrome P450 1A (EC 1.14.14.1) |
| CYP1A2 | Cytochrome P450 1A2 (EC 1.14.14.1) (CYPIA2) (Cholesterol 25-hydroxylase) (Cytochrome P(3)450) (Cytochrome P450 4) (Cytochrome P450-P3) (Hydroperoxy icosatetraenoate dehydratase) (EC 4.2.1.152) |
| CYP1B1 | Cytochrome P450 1B1 (EC 1.14.14.1) (CYPIB1) (Hydroperoxy icosatetraenoate dehydratase) (EC 4.2.1.152) |
| CYP21A2 | Cytochrome P450 family 21 subfamily A member 2 |
| CYP2A6 | Cytochrome P450 family 2 subfamily A member 6 |
| CYP2B6 | Cytochrome P450 family 2 subfamily B member 6 |
| CYP2C19 | Cytochrome P450 family 2 subfamily C member 19 |
| CYP2C9 | Cytochrome P450 family 2 subfamily C member 9 |
| CYP2D6 | Nonfunctional cytochrome P450 family 2 subfamily D polypeptide 6 (EC 1.14.14.1) |
| CYP2E1 | Cytochrome P450 family 2 subfamily E member 1 |
| CYP3A4 | Cytochrome P450 family 3 subfamily A member 4 |
| CYP51A1 | Cytochrome P450 family 51 subfamily A member 1 |
| DAB2IP | DAB2 interacting protein |
| DAD1 | Dolichyl-diphosphooligosaccharide--protein glycosyltransferase subunit DAD1 (Oligosaccharyl transferase subunit DAD1) (Defender against cell death 1) (DAD-1) |
| DAPK1 | Death-associated protein kinase beta |
| DAXX | Death domain associated protein |
| DBH | Dopamine beta-hydroxylase (EC 1.14.17.1) |
| DCSTAMP | Dendritic cell-specific transmembrane protein (DC-STAMP) (hDC-STAMP) (Dendrocyte-expressed seven transmembrane protein) (IL-four-induced protein) (FIND) (Transmembrane 7 superfamily member 4) |
| DDHD1 | DDHD domain containing 1 |
| DDIT3 | DNA damage inducible transcript 3 |
| DDOST | Dolichyl-diphosphooligosaccharide--protein glycosyltransferase 48 kDa subunit (Oligosaccharyl transferase 48 kDa subunit) |
| DDRGK1 | DDRGK domain-containing protein 1 |
| DDX3X | DEAD-box helicase 3 X-linked |
| DEGS1 | Delta 4-desaturase, sphingolipid 1 |
| DELE1 | DAP3 binding cell death enhancer 1 |
| DERL1 | Derlin |
| DERL2 | Derlin 2 |
| DERL3 | Derlin 3 |
| DES | Desmin |
| DGAT1 | Diacylglycerol O-acyltransferase 1 |
| DGAT2 | Diacylglycerol O-acyltransferase 2 |
| DHCR24 | 24-dehydrocholesterol reductase |
| DHCR7 | 7-dehydrocholesterol reductase |
| DHDDS | Alkyl transferase (EC 2.5.1.-) |
| DHX36 | ATP-dependent DNA/RNA helicase DHX36 |
| DICER1 | Endoribonuclease Dicer |
| DLAT | Dihydrolipoamide S-acetyltransferase |
| DLD | Dihydrolipoamide dehydrogenase |
| DLG1 | Discs large MAGUK scaffold protein 1 |
| DMD | Dystrophin |
| DMPK | DM1 protein kinase |
| DNAH8 | Dynein axonemal heavy chain 8 |
| DNAJA1 | DnaJ homolog subfamily A member 1 (DnaJ protein homolog 2) (HSDJ) (Heat shock 40 kDa protein 4) (Heat shock protein J2) (HSJ-2) (Human DnaJ protein 2) (hDj-2) |
| DNAJB1 | DnaJ homolog subfamily B member 1 (DnaJ protein homolog 1) (Heat shock 40 kDa protein 1) (HSP40) (Heat shock protein 40) (Human DnaJ protein 1) (hDj-1) |
| DNAJB11 | DnaJ heat shock protein family (Hsp40) member B11 |
| DNAJB12 | DnaJ heat shock protein family (Hsp40) member B12 |
| DNAJB14 | DnaJ heat shock protein family (Hsp40) member B14 |
| DNAJB2 | DnaJ heat shock protein family (Hsp40) member B2 |
| DNAJB9 | DNAJB9 protein (DnaJ (Hsp40) homolog, subfamily B, member 9) (DnaJ (Hsp40) homolog, subfamily B, member 9, isoform CRA_b) (MSTP049) (cDNA FLJ34885 fis, clone NT2NE2016608, highly similar to DnaJ homolog subfamily B member 9) |
| DNAJC10 | DnaJ heat shock protein family (Hsp40) member C10 |
| DNAJC18 | DnaJ heat shock protein family (Hsp40) member C18 |
| DNAJC3 | DnaJ heat shock protein family (Hsp40) member C3 |
| DNM1L | Dynamin 1 like |
| DPAGT1 | Dolichyl-phosphate N-acetylglucosaminephosphotransferase 1 |
| DPM1 | Dolichol-phosphate mannosyltransferase subunit 1 (EC 2.4.1.83) (Dolichol-phosphate mannose synthase subunit 1) (DPM synthase subunit 1) (Dolichyl-phosphate beta-D-mannosyltransferase subunit 1) (Mannose-P-dolichol synthase subunit 1) (MPD synthase subunit 1) |
| DPM2 | Dolichol phosphate-mannose biosynthesis regulatory protein |
| DPM3 | Dolichol-phosphate mannosyltransferase subunit 3 |
| DRD1 | D(1A) dopamine receptor (Dopamine D1 receptor) |
| DRD2 | D(2) dopamine receptor (Dopamine D2 receptor) |
| DSP | Desmoplakin |
| DSPP | Dentin sialophosphoprotein |
| DST | Dystonin |
| DUOXA1 | Dual oxidase maturation factor 1 alpha |
| DUSP19 | Dual specificity protein phosphatase 19 (EC 3.1.3.16) (EC 3.1.3.48) (Dual specificity phosphatase TS-DSP1) (Low molecular weight dual specificity phosphatase 3) (LMW-DSP3) (Protein phosphatase SKRP1) (Stress-activated protein kinase pathway-regulating phosphatase 1) (SAPK pathway-regulating phosphatase 1) |
| DYNC1H1 | Dynein cytoplasmic 1 heavy chain 1 |
| DYRK1A | Dual specificity tyrosine phosphorylation regulated kinase 1A |
| DYSF | Dysferlin |
| E2F1 | Transcription factor E2F1 (E2F-1) (PBR3) (Retinoblastoma-associated protein 1) (RBAP-1) (Retinoblastoma-binding protein 3) (RBBP-3) (pRB-binding protein E2F-1) |
| EBP | 3-beta-hydroxysteroid-Delta(8),Delta(7)-isomerase (EC 5.3.3.5) (Cholestenol Delta-isomerase) (Delta(8)-Delta(7) sterol isomerase) (D8-D7 sterol isomerase) (Emopamil-binding protein) |
| ECPAS | Ecm29 proteasome adaptor and scaffold |
| EDEM1 | ER degradation enhancing alpha-mannosidase like protein 1 |
| EDEM2 | ER degradation-enhancing alpha-mannosidase-like protein 2 |
| EDEM3 | ER degradation enhancing alpha-mannosidase like protein 3 |
| EDN1 | Endothelin-1 (Preproendothelin-1) (PPET1) [Cleaved into: Endothelin-1 (ET-1); Big endothelin-1] |
| EEF1A1 | Elongation factor 1-alpha |
| EEF1B2 | Elongation factor 1-beta |
| EEF1D | Elongation factor 1-delta |
| EEF2 | Eukaryotic translation elongation factor 2 |
| EGF | Epidermal growth factor |
| EGFR | Epidermal growth factor receptor |
| EGR1 | Early growth response protein 1 (EGR-1) (AT225) (Nerve growth factor-induced protein A) (NGFI-A) (Transcription factor ETR103) (Transcription factor Zif268) (Zinc finger protein 225) (Zinc finger protein Krox-24) |
| EIF2AK1 | Eukaryotic translation initiation factor 2 alpha kinase 1 |
| EIF2AK2 | Eukaryotic translation initiation factor 2 alpha kinase 2 |
| EIF2AK3 | Eukaryotic translation initiation factor 2 alpha kinase 3 |
| EIF2AK4 | Eukaryotic translation initiation factor 2 alpha kinase 4 |
| EIF2B1 | Translation initiation factor eIF-2B subunit alpha (eIF-2B GDP-GTP exchange factor subunit alpha) |
| EIF2B5 | Translation initiation factor eIF-2B subunit epsilon (eIF-2B GDP-GTP exchange factor subunit epsilon) |
| EIF2S1 | Eukaryotic translation initiation factor 2 subunit 1 (Eukaryotic translation initiation factor 2 subunit alpha) |
| EIF4E | Eukaryotic translation initiation factor 4E |
| EIF4G1 | Eukaryotic translation initiation factor 4 gamma 1 |
| EIF5A | Eukaryotic translation initiation factor 5A |
| ELAVL1 | ELAV like RNA binding protein 1 |
| ELAVL4 | ELAV like RNA binding protein 4 |
| ELN | Elastin (Tropoelastin) |
| ELOVL1 | Elongation of very long chain fatty acids protein 1 (EC 2.3.1.199) (3-keto acyl-CoA synthase ELOVL1) (ELOVL fatty acid elongase 1) (ELOVL FA elongase 1) (Very long chain 3-ketoacyl-CoA synthase 1) (Very long chain 3-oxoacyl-CoA synthase 1) |
| ELOVL2 | Elongation of very long chain fatty acids protein 2 (EC 2.3.1.199) (3-keto acyl-CoA synthase ELOVL2) (ELOVL fatty acid elongase 2) (ELOVL FA elongase 2) (Very long chain 3-ketoacyl-CoA synthase 2) (Very long chain 3-oxoacyl-CoA synthase 2) |
| ELOVL3 | Elongation of very long chain fatty acids protein 3 (EC 2.3.1.199) (3-keto acyl-CoA synthase ELOVL3) (Cold-inducible glycoprotein of 30 kDa) (ELOVL fatty acid elongase 3) (ELOVL FA elongase 3) (Very long chain 3-ketoacyl-CoA synthase 3) (Very long chain 3-oxoacyl-CoA synthase 3) |
| ELOVL4 | Elongation of very long chain fatty acids protein 4 (EC 2.3.1.199) (3-keto acyl-CoA synthase ELOVL4) (ELOVL fatty acid elongase 4) (ELOVL FA elongase 4) (Very long chain 3-ketoacyl-CoA synthase 4) (Very long chain 3-oxoacyl-CoA synthase 4) |
| ELOVL5 | Elongation of very long chain fatty acids protein 5 (EC 2.3.1.199) (3-keto acyl-CoA synthase ELOVL5) (ELOVL fatty acid elongase 5) (ELOVL FA elongase 5) (Very long chain 3-ketoacyl-CoA synthase 5) (Very long chain 3-oxoacyl-CoA synthase 5) |
| ELOVL7 | Elongation of very long chain fatty acids protein 7 (EC 2.3.1.199) (3-keto acyl-CoA synthase ELOVL7) (ELOVL fatty acid elongase 7) (ELOVL FA elongase 7) (Very long chain 3-ketoacyl-CoA synthase 7) (Very long chain 3-oxoacyl-CoA synthase 7) |
| EMC1 | ER membrane protein complex subunit 1 |
| EMC10 | ER membrane protein complex subunit 10 |
| EMC2 | ER membrane protein complex subunit 2 |
| EMC3 | ER membrane protein complex subunit 3 (Transmembrane protein 111) |
| EMC4 | ER membrane protein complex subunit 4 |
| EMC6 | ER membrane protein complex subunit 6 (Transmembrane protein 93) |
| EMC7 | ER membrane protein complex subunit 7 |
| EMC8 | ER membrane protein complex subunit 8 |
| EMC9 | ER membrane protein complex subunit 9 |
| EMD | Emerin |
| ENPP1 | Ectonucleotide pyrophosphatase/phosphodiesterase 1 |
| ENTPD5 | Ectonucleoside triphosphate diphosphohydrolase 5 (inactive) |
| EOGT | EGF domain specific O-linked N-acetylglucosamine transferase |
| EP300 | E1A binding protein p300 |
| EPAS1 | Endothelial PAS domain protein 1 |
| EPHX1 | Epoxide hydrolase 1 microsomal isoform 2 |
| EPM2A | EPM2A glucan phosphatase, laforin |
| EPO | Erythropoietin (Epoetin) |
| ERAP1 | Endoplasmic reticulum aminopeptidase-1 |
| ERAP2 | Endoplasmic reticulum aminopeptidase 2 |
| ERBB2 | Erb-b2 receptor tyrosine kinase 2 (cDNA FLJ59426, highly similar to Receptor tyrosine-protein kinase erbB-2) |
| ERGIC2 | Endoplasmic reticulum-Golgi intermediate compartment protein |
| ERGIC3 | Endoplasmic reticulum-Golgi intermediate compartment protein |
| ERLEC1 | Endoplasmic reticulum lectin |
| ERLIN1 | Erlin-1 (Endoplasmic reticulum lipid raft-associated protein 1) (Protein KE04) (Stomatin-prohibitin-flotillin-HflC/K domain-containing protein 1) (SPFH domain-containing protein 1) |
| ERLIN2 | Erlin-2 (Endoplasmic reticulum lipid raft-associated protein 2) (Stomatin-prohibitin-flotillin-HflC/K domain-containing protein 2) (SPFH domain-containing protein 2) |
| ERMARD | ER membrane associated RNA degradation (Endoplasmic reticulum membrane-associated RNA degradation protein) |
| ERMP1 | Endoplasmic reticulum metallopeptidase 1 |
| ERN1 | Endoplasmic reticulum to nucleus signaling 1 |
| ERN2 | Endoplasmic reticulum to nucleus signaling 2 |
| ERO1A | Endoplasmic reticulum oxidoreductase 1 alpha |
| ERO1B | Endoplasmic reticulum oxidoreductase 1 beta |
| ERP27 | Endoplasmic reticulum protein 27 |
| ERP29 | Endoplasmic reticulum resident protein 29 |
| ERP44 | Endoplasmic reticulum protein 44 |
| ESR1 | Estrogen receptor 1 |
| ESYT1 | Extended synaptotagmin 1 |
| ESYT2 | Extended synaptotagmin 2 |
| EXT1 | Exostosin glycosyltransferase 1 |
| EZH2 | Enhancer of zeste 2 polycomb repressive complex 2 subunit |
| F10 | Coagulation factor X |
| F2 | Coagulation factor II, thrombin |
| F3 | Tissue factor (TF) (Coagulation factor III) (Thromboplastin) (CD antigen CD142) |
| F5 | Coagulation factor V |
| F7 | Coagulation factor VII |
| F8 | Coagulation factor VIII |
| F9 | Coagulation factor IX |
| FAAH | Fatty acid amide hydrolase |
| FAF1 | Fas associated factor 1 (cDNA FLJ37524 fis, clone BRCAN2007119, highly similar to FAS-associated factor 1) |
| FAF2 | Fas associated factor family member 2 |
| FAM120A | Family with sequence similarity 120A |
| FAM8A1 | Family with sequence similarity 8 member A1 |
| FANCD2 | FA complementation group D2 |
| FAS | Fas cell surface death receptor |
| FASLG | Tumor necrosis factor ligand superfamily member 6 (CD95 ligand) (Fas antigen ligand) |
| FBN1 | Fibrillin-1 preproprotein |
| FBXO17 | F-box protein 17 |
| FBXO2 | F-box protein 2 |
| FBXO27 | F-box protein 27 |
| FBXO44 | F-box protein 44 |
| FBXO6 | F-box protein 6 |
| FCGR2B | Fc-gamma-receptor IIB |
| FGF2 | Fibroblast growth factor (FGF) |
| FGF21 | Fibroblast growth factor 21 (FGF-21) |
| FGFR3 | Fibroblast growth factor receptor 3 |
| FGFR4 | Fibroblast growth factor receptor 4 |
| FICD | FIC domain protein adenylyltransferase |
| FITM2 | Acyl-coenzyme A diphosphatase FITM2 (EC 3.6.1.-) (Fat storage-inducing transmembrane protein 2) (Fat-inducing protein 2) |
| FKBP10 | peptidylprolyl isomerase (EC 5.2.1.8) |
| FKBP14 | Peptidyl-prolyl cis-trans isomerase FKBP14 (PPIase FKBP14) (EC 5.2.1.8) (22 kDa FK506-binding protein) (22 kDa FKBP) (FKBP-22) (FK506-binding protein 14) (FKBP-14) (Rotamase) |
| FKBP1A | peptidylprolyl isomerase (EC 5.2.1.8) |
| FKBP1B | peptidylprolyl isomerase (EC 5.2.1.8) |
| FKBP4 | peptidylprolyl isomerase (EC 5.2.1.8) |
| FKBP5 | peptidylprolyl isomerase (EC 5.2.1.8) |
| FKRP | Fukutin related protein |
| FLNB | Filamin B |
| FLOT1 | Flotillin-1 |
| FLT3 | Fms-like tyrosine kinase 3 |
| FMR1 | Fragile X messenger ribonucleoprotein 1 |
| FN1 | Fibronectin 1 |
| FOS | Fos proto-oncogene, AP-1 transcription factor subunit |
| FOXO1 | Alternative protein FOXO1 |
| FOXO3 | Forkhead box protein O3 (AF6q21 protein) (Forkhead in rhabdomyosarcoma-like 1) |
| FOXRED2 | FAD dependent oxidoreductase domain containing 2 |
| FURIN | Furin, paired basic amino acid cleaving enzyme |
| FUS | Fusion (Involved in t(1216) in malignant liposarcoma) |
| G3BP1 | G3BP stress granule assembly factor 1 |
| G3BP2 | G3BP stress granule assembly factor 2 |
| G6PC1 | Glucose-6-phosphatase catalytic subunit 1 |
| G6PC2 | Glucose-6-phosphatase 2 (Glucose-6-phosphatase catalytic subunit 2) |
| G6PC3 | Glucose-6-phosphatase catalytic subunit 3 |
| G6PD | Glucose-6-phosphate dehydrogenase |
| GABARAP | GABA type A receptor-associated protein |
| GABARAPL1 | GABA type A receptor associated protein like 1 |
| GABARAPL2 | GABA type A receptor associated protein like 2 |
| GABRA1 | Gamma-aminobutyric acid type A receptor subunit alpha1 |
| GADD45A | Growth arrest and DNA damage inducible alpha |
| GANAB | Glucosidase II alpha subunit |
| GAPDH | Glyceraldehyde-3-phosphate dehydrogenase |
| GATA1 | GATA binding protein 1 |
| GBA | Glucosylceramidase (EC 3.2.1.45) |
| GBA2 | Glucosylceramidase beta 2 |
| GBF1 | Golgi brefeldin A resistant guanine nucleotide exchange factor 1 |
| GCH1 | GTP cyclohydrolase 1 (EC 3.5.4.16) (GTP cyclohydrolase I) |
| GCLC | Glutamate-cysteine ligase catalytic subunit |
| GDF15 | Growth/differentiation factor 15 |
| GET1 | Guided entry of tail-anchored proteins factor 1 |
| GET3 | Guided entry of tail-anchored proteins factor 3 |
| GET4 | Guided entry of tail-anchored proteins factor 4 |
| GFAP | Glial fibrillary acidic protein |
| GFPT1 | Glutamine--fructose-6-phosphate transaminase 1 |
| GH1 | Growth hormone 1 variant 1 |
| GH-LCR | GH-LCR |
| GHRL | Ghrelin and obestatin prepropeptide |
| GJA1 | Gap junction alpha-1 protein (Connexin-43) (Cx43) (Gap junction 43 kDa heart protein) |
| GLA | Alpha-galactosidase (EC 3.2.1.-) |
| GLUD1 | Glutamate dehydrogenase 1 |
| GOLGA2 | Golgin subfamily A member 2 |
| GOLGB1 | Golgin subfamily B member 1 (372 kDa Golgi complex-associated protein) (GCP372) (Giantin) (Macrogolgin) |
| GOLPH3 | Golgi phosphoprotein 3 |
| GORASP1 | Golgi reassembly stacking protein 1 |
| GORASP2 | Golgi reassembly stacking protein 2 |
| GOSR1 | Golgi SNAP receptor complex member 1 |
| GOSR2 | Golgi SNAP receptor complex member 2 |
| GPAA1 | Glycosylphosphatidylinositol anchor attachment 1 |
| GPAT3 | Glycerol-3-phosphate acyltransferase 3 (GPAT-3) (EC 2.3.1.15) (1-acyl-sn-glycerol-3-phosphate O-acyltransferase 10) (AGPAT 10) (1-acyl-sn-glycerol-3-phosphate O-acyltransferase 9) (1-AGP acyltransferase 9) (1-AGPAT 9) (EC 2.3.1.51) (Acyl-CoA:glycerol-3-phosphate acyltransferase 3) (hGPAT3) (Lung cancer metastasis-associated protein 1) (Lysophosphatidic acid acyltransferase theta) (LPAAT-theta) (MAG-1) |
| GPER1 | G protein-coupled estrogen receptor 1 |
| GPI | Glucose-6-phosphate isomerase |
| GPR37 | Prosaposin receptor GPR37 (Endothelin B receptor-like protein 1) (ETBR-LP-1) (G-protein coupled receptor 37) (Parkin-associated endothelin receptor-like receptor) (PAELR) |
| GPX1 | Glutathione peroxidase 1 |
| GPX7 | Glutathione peroxidase 7 (GPx-7) (GSHPx-7) (EC 1.11.1.9) (CL683) |
| GPX8 | Glutathione peroxidase 8 (putative) |
| GRAMD1A | GRAM domain containing 1A |
| GRAMD1B | GRAM domain containing 1B |
| GRIA1 | Glutamate receptor 1 variant |
| GRIN1 | Glutamate ionotropic receptor NMDA type subunit 1 |
| GRIN2A | Glutamate ionotropic receptor NMDA type subunit 2A |
| GRIN2B | Glutamate ionotropic receptor NMDA type subunit 2B |
| GRINA | Glutamate ionotropic receptor NMDA type subunit associated protein 1 |
| GRP | Gastrin-releasing peptide nirs variant 1 |
| GSK3B | Glycogen synthase kinase 3 beta |
| GSR | Glutathione-disulfide reductase |
| GSTM1 | Glutathione S-transferase mu 1 |
| GSTP1 | Glutathione S-transferase pi 1 |
| GTF2I | General transcription factor II-I |
| H2AX | Histone H2AX (H2a/x) (Histone H2A.X) |
| H6PD | GDH/6PGL endoplasmic bifunctional protein [Includes: Hexose-6-phosphate dehydrogenase (Glucose 1-dehydrogenase) (GDH) (EC 1.1.1.47) (Glucose-6-phosphate dehydrogenase) (EC 1.1.1.363); 6-phosphogluconolactonase (6PGL) (EC 3.1.1.31)] |
| HACD2 | Very-long-chain (3R)-3-hydroxyacyl-CoA dehydratase (EC 4.2.1.134) |
| HACD3 | Very-long-chain (3R)-3-hydroxyacyl-CoA dehydratase (EC 4.2.1.134) |
| HACE1 | HECT domain and ankyrin repeat containing E3 ubiquitin protein ligase 1 |
| HADHB | Hydroxyacyl-CoA dehydrogenase trifunctional multienzyme complex subunit beta |
| HAX1 | HCLS1 associated protein X-1 |
| HCRT | Hypocretin neuropeptide precursor (Hypocretin) (Hcrt) (Orexin precursor) (Prepro-orexin) (Preprohypocretin) [Cleaved into: Orexin-A (Hypocretin-1) (Hcrt1); Orexin-B (Hypocretin-2) (Hcrt2)] |
| HDAC6 | Histone deacetylase 6 |
| HERPUD1 | Homocysteine inducible ER protein with ubiquitin like domain 1 |
| HERPUD2 | HERPUD family member 2 |
| HFE | Homeostatic iron regulator |
| HGSNAT | Heparan-alpha-glucosaminide N-acetyltransferase |
| HIF1A | Hypoxia-inducible factor 1-alpha |
| HLA-A | Leucocyte antigen class I |
| HLA-B | HLA class 1 antigen |
| HLA-C | MHC class I antigen |
| HLA-DPB1 | MHC class II antigen |
| HLA-DRA | HLA class II histocompatibility antigen, DR alpha chain |
| HLA-DRB1 | HLA class II histocompatibility antigen, DRB1 beta chain (Human leukocyte antigen DRB1) (HLA-DRB1) |
| HLA-G | HLA class I histocompatibility antigen, alpha chain G |
| HM13 | Histocompatibility minor 13 |
| HMGB1 | High mobility group box 1 |
| HMGCLL1 | 3-hydroxymethyl-3-methylglutaryl-CoA lyase like 1 |
| HMGCR | 3-hydroxy-3-methylglutaryl-CoA reductase |
| HMOX1 | Heme oxygenase 1 |
| HMOX2 | Heme oxygenase 2 |
| HNF4A | Hepatocyte nuclear factor 4 alpha |
| HNRNPK | Heterogeneous nuclear ribonucleoprotein K |
| HNRNPU | Heterogeneous nuclear ribonucleoprotein U |
| HP | Haptoglobin |
| HRAS | GTPase HRas |
| HRC | Histidine rich calcium binding protein |
| HSD11B1 | Hydroxysteroid 11-beta dehydrogenase 1 |
| HSD11B2 | Hydroxysteroid 11-beta dehydrogenase 2 |
| HSD17B10 | Hydroxysteroid 17-beta dehydrogenase 10 |
| HSD17B12 | Hydroxysteroid 17-beta dehydrogenase 12 |
| HSF1 | Heat shock transcription factor 1 |
| HSP90AA1 | Heat shock protein 90 alpha family class A member 1 |
| HSP90AB1 | Heat shock protein 90kDa alpha (Cytosolic), class B member 1, isoform CRA_a |
| HSP90B1 | Heat shock protein 90 beta family member 1 |
| HSPA13 | Heat shock 70 kDa protein 13 (Microsomal stress-70 protein ATPase core) (Stress-70 protein chaperone microsome-associated 60 kDa protein) |
| HSPA1A | Heat shock 70 kDa protein 1A (Heat shock protein family A (Hsp70) member 1A) |
| HSPA1B | Heat shock 70 kDa protein 1B (Heat shock 70 kDa protein 2) (HSP70-2) (HSP70.2) |
| HSPA1L | Heat shock 70 kDa protein 1-like (Heat shock 70 kDa protein 1L) (Heat shock 70 kDa protein 1-Hom) (HSP70-Hom) |
| HSPA2 | Heat shock-related 70 kDa protein 2 (Heat shock 70 kDa protein 2) |
| HSPA4 | Heat shock 70 kDa protein 4 (HSP70RY) (Heat shock 70-related protein APG-2) |
| HSPA5 | 78 kDa glucose-regulated protein (Binding-immunoglobulin protein) (Heat shock protein 70 family protein 5) (Heat shock protein family A member 5) (Immunoglobulin heavy chain-binding protein) |
| HSPA6 | Heat shock 70 kDa protein 6 (Heat shock 70 kDa protein B') |
| HSPA8 | Heat shock protein family A (Hsp70) member 8 |
| HSPA9 | Heat shock protein family A (Hsp70) member 9 |
| HSPB1 | Heat shock protein family B (small) member 1 |
| HSPD1 | Heat shock 60kDa protein 1 isoform 4 |
| HSPG2 | Heparan sulfate proteoglycan 2 |
| HTR1A | 5-hydroxytryptamine receptor 1A |
| HTR2A | 5-hydroxytryptamine receptor 2A (Serotonin receptor 2A) |
| HTR3A | 5-hydroxytryptamine receptor 3A |
| HTRA2 | Serine protease HTRA2, mitochondrial |
| HTT | Huntingtin isoform A |
| HUWE1 | Alternative protein HUWE1 |
| HYOU1 | Hypoxia up-regulated protein 1 |
| IAPP | Islet amyloid polypeptide |
| ICAM1 | Intercellular adhesion molecule 1 |
| ICMT | Isoprenylcysteine carboxyl methyltransferase |
| IER3IP1 | Immediate early response 3-interacting protein 1 |
| IFNG | Interferon gamma (IFN-gamma) |
| IGF1 | Insulin-like growth factor-I |
| IGF1R | Insulin like growth factor 1 receptor |
| IGF2BP1 | Alternative protein IGF2BP1 |
| IKBKG | NF-kappa-B essential modulator |
| IL10 | Interleukin family protein |
| IL15RA | Interleukin 15 receptor subunit alpha |
| IL18 | Interleukin-18 (IL-18) |
| IL1A | Interleukin-1 alpha (IL-1 alpha) (Hematopoietin-1) |
| IL1B | Multifunctional fusion protein [Includes: Interleukin-1; Interleukin-1 beta] |
| IL1RN | Interleukin 1 receptor antagonist |
| IL2 | Interleukin-2 (IL-2) |
| IL6 | Truncated interleukin 6 |
| ILVBL | IlvB acetolactate synthase like |
| INPP5K | Inositol polyphosphate-5-phosphatase K |
| INS | Insulin |
| INSIG1 | Insulin-induced gene 1 protein (INSIG-1) |
| INSIG2 | Insulin-induced gene 2 protein (INSIG-2) |
| INSR | Insulin receptor |
| ITGB1 | Integrin subunit beta 1 |
| ITPR1 | Inositol 1,4,5-trisphosphate receptor type 1 |
| ITPR2 | Inositol 1,4,5-trisphosphate receptor type 2 |
| ITPR3 | Inositol 1,4,5-trisphosphate receptor type 3 (IP3 receptor isoform 3) (IP3R 3) (InsP3R3) (Type 3 inositol 1,4,5-trisphosphate receptor) (Type 3 InsP3 receptor) |
| JAGN1 | Protein jagunal homolog 1 |
| JAK2 | Janus kinase 2 |
| JKAMP | JNK1/MAPK8 associated membrane protein |
| JPH1 | Junctophilin 1 |
| JPH2 | JPH2 protein |
| JPH3 | Junctophilin-3 |
| JPH4 | Junctophilin 4 |
| JSRP1 | Junctional sarcoplasmic reticulum protein 1 (Junctional-face membrane protein of 45 kDa homolog) (JP-45) |
| JUN | Junctional sarcoplasmic reticulum protein 1 (Junctional-face membrane protein of 45 kDa homolog) (JP-45) |
| KCNA2 | Potassium voltage-gated channel subfamily A member 2 |
| KCNB1 | Potassium voltage-gated channel subfamily B member 1 |
| KCNE1 | Potassium voltage-gated channel Isk-related family member 1 |
| KCNE2 | Cardiac voltage-gated potassium channel accessory subunit 2 |
| KCNH2 | Voltage-gated channel subfamily H member 2 |
| KCNIP4 | Potassium voltage-gated channel interacting protein 4 |
| KCNJ11 | Potassium inwardly rectifying channel subfamily J member 11 |
| KCNJ2 | Inward rectifier potassium channel 2 (Cardiac inward rectifier potassium channel) (Inward rectifier K(+) channel Kir2.1) (IRK-1) (hIRK1) (Potassium channel, inwardly rectifying subfamily J member 2) |
| KCNJ5 | G protein-activated inward rectifier potassium channel 4 |
| KCNMA1 | Potassium calcium-activated channel subfamily M alpha 1 |
| KCNN4 | Potassium calcium-activated channel subfamily N member 4 |
| KCNQ1 | Potassium voltage-gated channel subfamily Q member 1 |
| KCNQ2 | Potassium voltage-gated channel subfamily Q member 2 |
| KDELR1 | KDEL endoplasmic reticulum protein retention receptor 1 |
| KDELR2 | KDEL endoplasmic reticulum protein retention receptor 2 |
| KDELR3 | ER lumen protein-retaining receptor 3 (KDEL endoplasmic reticulum protein retention receptor 3) (KDEL receptor 3) |
| KDR | Kinase insert domain receptor (EC 2.7.10.1) |
| KDSR | 3-ketodihydrosphingosine reductase |
| KEAP1 | Kelch-like ECH-associated protein 1 |
| KIF1C | Kinesin family member 1C |
| KL | Klotho (EC 3.2.1.31) [Cleaved into: Klotho peptide] |
| KMT2B | Lysine methyltransferase 2B |
| KNG1 | Kininogen 1, isoform CRA_a |
| KPNA2 | Karyopherin subunit alpha 2 |
| KPNB1 | Karyopherin subunit beta 1 |
| KRAS | K-Ras |
| KRT8 | Keratin 8 |
| KRTCAP2 | Keratinocyte-associated protein 2 (Dolichyl-diphosphooligosaccharide--protein glycosyltransferase subunit KCP2) |
| KTN1 | Kinectin 1 |
| LACC1 | Laccase domain containing 1 |
| LAMA2 | Laminin subunit alpha 2 |
| LAMP1 | Lysosome-associated membrane glycoprotein 1 (LAMP-1) (Lysosome-associated membrane protein 1) (CD107 antigen-like family member A) (CD antigen CD107a) |
| LAMP2 | Lysosome-associated membrane glycoprotein 2 (LAMP-2) (Lysosome-associated membrane protein 2) (CD107 antigen-like family member B) (LGP-96) (CD antigen CD107b) |
| LBR | Lamin B receptor |
| LDHA | Lactate dehydrogenase A |
| LDLR | Low-density lipoprotein receptor |
| LEP | Leptin (Obesity factor) |
| LGALS1 | Lectin, galactoside-binding, soluble, 1 |
| LGALS3 | Galectin 3 |
| LGI4 | Leucine rich repeat LGI family member 4 |
| LIN28A | Protein lin-28 homolog A (Lin-28A) (Zinc finger CCHC domain-containing protein 1) |
| LIPC | Hepatic lipase |
| LMAN1 | LMAN1 protein |
| LMAN1L | Lectin, mannose binding 1 like |
| LMAN2 | Lectin, mannose binding 2 |
| LMBRD1 | LMBR1 domain containing 1 |
| LMNA | Prelamin-A/C (Rhabdomyosarcoma antigen MU-RMS-40.12) |
| LMNB1 | Alternative protein LMNB1 |
| LNPK | Lunapark, ER junction formation factor |
| LOC110806262 | LOC110806262 |
| LONP1 | Lon peptidase 1, mitochondrial |
| LPCAT1 | Lysophosphatidylcholine acyltransferase 1 |
| LPCAT3 | Lysophosphatidylcholine acyltransferase 3 |
| LPL | Lipoprotein lipase |
| LRIT3 | Leucine rich repeat, Ig-like and transmembrane domains 3 |
| LRP2 | LDL receptor related protein 2 |
| LRP6 | Low-density lipoprotein receptor-related protein |
| LRPAP1 | LDL receptor related protein associated protein 1 |
| LRRC59 | Leucine rich repeat containing 59 |
| LRRK2 | Leucine rich repeat kinase 2 |
| LSG1 | Large 60S subunit nuclear export GTPase 1 |
| MAGEA3 | MAGE family member A3 |
| MAN1A1 | Mannosyl-oligosaccharide 1,2-alpha-mannosidase IA (EC 3.2.1.113) (Man(9)-alpha-mannosidase) (Man9-mannosidase) (Mannosidase alpha class 1A member 1) (Processing alpha-1,2-mannosidase IA) (Alpha-1,2-mannosidase IA) |
| MAN1B1 | Mannosidase alpha class 1B member 1 |
| MAN2B1 | Mannosidase alpha class 2B member 1 |
| MANF | Mesencephalic astrocyte derived neurotrophic factor |
| MAOA | Monoamine oxidase A |
| MAOB | Amine oxidase (EC 1.4.3.-) |
| MAP1LC3A | Microtubule-associated proteins 1A/1B light chain 3A (Autophagy-related protein LC3 A) (Autophagy-related ubiquitin-like modifier LC3 A) (MAP1 light chain 3-like protein 1) (MAP1A/MAP1B light chain 3 A) (MAP1A/MAP1B LC3 A) (Microtubule-associated protein 1 light chain 3 alpha) |
| MAP1LC3B | Microtubule-associated proteins 1A/1B light chain 3B (Autophagy-related protein LC3 B) (Autophagy-related ubiquitin-like modifier LC3 B) (MAP1 light chain 3-like protein 2) (MAP1A/MAP1B light chain 3 B) (MAP1A/MAP1B LC3 B) (Microtubule-associated protein 1 light chain 3 beta) |
| MAP2K1 | Mitogen-activated protein kinase kinase 1 |
| MAP2K3 | Mitogen activated protein kinase kinase 3 |
| MAP2K4 | Mitogen-activated protein kinase kinase 4 |
| MAP2K6 | Mitogen-activated protein kinase kinase 6 |
| MAP2K7 | Mitogen-activated protein kinase kinase kinase 7 |
| MAP3K5 | mitogen-activated protein kinase kinase kinase (EC 2.7.11.25) |
| MAP3K7 | Mitogen-activated protein kinase kinase kinase 7 |
| MAPK1 | Mitogen-activated protein kinase (EC 2.7.11.24) |
| MAPK10 | Mitogen-activated protein kinase 10 |
| MAPK13 | Mitogen-activated protein kinase 13 |
| MAPK14 | Mitogen-activated protein kinase 14 |
| MAPK3 | Mitogen-activated protein kinase 3 |
| MAPK8 | Mitogen-activated protein kinase 8 |
| MAPK8IP1 | Mitogen-activated protein kinase 8 interacting protein 1 |
| MAPK9 | Mitogen-activated protein kinase 9 |
| MAPKAP1 | Target of rapamycin complex 2 subunit MAPKAP1 (Mitogen-activated protein kinase 2-associated protein 1) (Stress-activated map kinase-interacting protein 1) |
| MAPKAPK2 | MAP kinase-activated protein kinase 2 (MAPK-activated protein kinase 2) (MAPKAP kinase 2) (MAPKAP-K2) (MAPKAPK-2) (MK-2) (MK2) (EC 2.7.11.1) |
| MAPT | Microtubule-associated protein |
| MARCHF6 | Membrane associated ring-CH-type finger 6 |
| MARCKS | Myristoylated alanine-rich C-kinase substrate (MARCKS) (Protein kinase C substrate, 80 kDa protein, light chain) (80K-L protein) (PKCSL) |
| MATN3 | Matrilin-3 |
| MBTPS1 | Membrane bound transcription factor peptidase, site 1 |
| MBTPS2 | Membrane-bound transcription factor site-2 protease (EC 3.4.24.85) (Endopeptidase S2P) |
| MCFD2 | Multiple coagulation factor deficiency 2, ER cargo receptor complex subunit |
| MCL1 | Myosin light chain 1 |
| MDM2 | Methyl CpG binding protein 2 |
| MECP2 | Truncated methyl CpG binding protein 2 transcript 1 |
| MEF2A | Myocyte enhancer factor 2A isoform 4 |
| MEF2C | Myocyte enhancer factor 2C |
| MET | MET protein |
| MFN2 | Mitofusin 2 |
| MGAT2 | Alpha-1,6-mannosyl-glycoprotein 2-beta-N-acetylglucosaminyltransferase (EC 2.4.1.143) (Beta-1,2-N-acetylglucosaminyltransferase II) (GlcNAc-T II) (GNT-II) (Mannoside acetylglucosaminyltransferase 2) (N-glycosyl-oligosaccharide-glycoprotein N-acetylglucosaminyltransferase II) |
| MGST1 | Microsomal glutathione S-transferase 1 |
| MIA2 | MIA SH3 domain ER export factor 2 |
| MIA3 | MIA SH3 domain ER export factor 3 |
| MICA | MHC class I chain-related A antigen |
| MICB | MHC class I chain-related protein B |
| MIR199A1 | MIR199A1 |
| MIR200C | MIR200C |
| MIR21 | MIR21 |
| MIR34A | MIR34A |
| MLEC | Malectin |
| MMGT1 | Membrane magnesium transporter 1 |
| MMP2 | Matrix metallopeptidase 2 |
| MMP9 | Matrix metalloproteinase-9 (MMP-9) (EC 3.4.24.35) (92 kDa gelatinase) (92 kDa type IV collagenase) (Gelatinase B) (GELB) [Cleaved into: 67 kDa matrix metalloproteinase-9; 82 kDa matrix metalloproteinase-9] |
| MOGS | Mannosyl-oligosaccharide glucosidase |
| MOSPD2 | Motile sperm domain-containing protein 2 |
| MPO | Myeloperoxidase |
| MPPE1 | Metallophosphoesterase 1 |
| MR1 | Major histocompatibility complex class I-related gene protein (MHC class I-related gene protein) (Class I histocompatibility antigen-like protein) |
| MSRA | Mitochondrial peptide methionine sulfoxide reductase (EC 1.8.4.11) (Peptide-methionine (S)-S-oxide reductase) (Peptide Met(O) reductase) (Protein-methionine-S-oxide reductase) (PMSR) |
| MSRB1 | Methionine sulfoxide reductase B1 |
| MSRB3 | Methionine sulfoxide reductase B3 |
| MTDH | Metadherin |
| MTHFR | Methylenetetrahydrofolate reductase |
| MTOR | Mechanistic target of rapamycin kinase |
| MTTP | Microsomal triglyceride transfer protein |
| MUC1 | Mucin 1, cell surface associated |
| MUC5AC | Mucin 5AC, oligomeric mucus/gel-forming |
| MX1 | Interferon-induced GTP-binding protein Mx1 (Interferon-induced protein p78) (IFI-78K) (Interferon-regulated resistance GTP-binding protein MxA) (Myxoma resistance protein 1) (Myxovirus resistance protein 1) [Cleaved into: Interferon-induced GTP-binding protein Mx1, N-terminally processed] |
| MYC | C-myc protein |
| MYDGF | Myeloid derived growth factor |
| MYH6 | Myosin heavy chain 6 |
| MYH7 | Cardiac muscle myosin heavy chain 7 beta |
| MYLK | Myosin light chain kinase |
| MYO9A | Myosin IXA |
| MYOC | Mutant myocilin |
| MYRF | Myelin regulatory factor |
| MZB1 | Marginal zone B and B1 cell specific protein |
| NACA | Nascent polypeptide associated complex subunit alpha |
| NAGLU | N-acetyl-alpha-glucosaminidase |
| NAPA | NSF attachment protein alpha |
| NAT8 |  |
| NBAS | NBAS subunit of NRZ tethering complex (Neuroblastoma-amplified gene protein) (Neuroblastoma-amplified sequence) |
| NCCRP1 | F-box only protein 50 (NCC receptor protein 1 homolog) (NCCRP-1) (Non-specific cytotoxic cell receptor protein 1 homolog) |
| NCK1 | NCK adaptor protein 1 |
| NCK2 | NCK adaptor protein 2 |
| NCLN | Nicalin |
| NCSTN | Nicastrin |
| NDRG1 | N-myc downstream regulated 1 |
| NDUFS4 | NADH:ubiquinone oxidoreductase subunit S4 |
| NDUFS8 | NADH:ubiquinone oxidoreductase core subunit S8 |
| NEPRO | Nucleolus and neural progenitor protein |
| NF1 | Truncated neurofibromin 1 |
| NFE2L1 | NFE2 like bZIP transcription factor 1 |
| NFE2L2 | NFE2 like bZIP transcription factor 2 |
| NFKB1 | Nuclear factor kappa B subunit 1 |
| NGF | Beta-nerve growth factor |
| NGLY1 | Peptide-N(4)-(N-acetyl-beta-glucosaminyl)asparagine amidase (EC 3.5.1.52) (N-glycanase 1) (Peptide:N-glycanase) |
| NHLRC1 | E3 ubiquitin-protein ligase NHLRC1 (EC 2.3.2.27) (Malin) (NHL repeat-containing protein 1) (RING-type E3 ubiquitin transferase NHLRC1) |
| NIBAN1 | Niban apoptosis regulator 1 |
| NLRP1 | NLR family pyrin domain containing 1 |
| NLRP3 | NLR family pyrin domain containing 3 |
| NOD2 | Nucleotide binding oligomerization domain containing 2 |
| NOL3 | Nucleolar protein 3 |
| NOS1 | NOS1 protein |
| NOS1AP | Nitric oxide synthase 1 adaptor protein |
| NOS2 | Nitric oxide synthase 2 |
| NOS3 | Nitric oxide synthase 3 |
| NOTCH1 | Notch receptor 1 |
| NOTCH2 | Notch receptor 2 |
| NOTCH3 | Notch receptor 3 |
| NOX4 | NADPH oxidase 4 |
| NOX5 | NADPH oxidase 5 |
| NPC1 | Niemann-Pick C1 protein |
| NPC2 | NPC intracellular cholesterol transporter 2 |
| NPLOC4 | NPL4 homolog, ubiquitin recognition factor |
| NPM1 | Nucleophosmin |
| NPPA | Natriuretic peptides A (Atrial natriuretic factor prohormone) (Atrial natriuretic peptide prohormone) (Atriopeptigen) (Cardiodilatin) (preproCDD-ANF) |
| NPPB | Natriuretic peptides B (Brain natriuretic factor prohormone) (preproBNP) (proBNP) (Gamma-brain natriuretic peptide) (Iso-ANP) [Cleaved into: NT-proBNP (NT-pro-BNP) (NT-proBNP(1-76)); proBNP(3-108); Brain natriuretic peptide 32 (BNP(1-32)) (BNP-32) (Brain natriuretic peptide) (BNP); BNP(1-30); BNP(1-29); BNP(1-28); BNP(2-31); BNP(3-32) (des-SerPro-BNP) (proBNP(79-108)); BNP(3-30); BNP(3-29); Brain natriuretic peptide 29 (BNP(4-32)); BNP(4-31); BNP(4-30); BNP(4-29); BNP(4-27); BNP(5-32); BNP(5-31); BNP(5-29)] |
| NPY | Pro-neuropeptide Y [Cleaved into: Neuropeptide Y (Neuropeptide tyrosine) (NPY); C-flanking peptide of NPY (CPON)] |
| NQO1 | NAD(P)H quinone dehydrogenase 1 |
| NR1H2 | Liver X receptor beta |
| NR1H3 | Liver X receptor alpha |
| NR3C1 | Glucocorticoid receptor (Nuclear receptor subfamily 3 group C member 1) |
| NR3C2 | Mineralocorticoid receptor (Nuclear receptor subfamily 3, group C, member 2 variant 1) |
| NR4A1 | Nuclear receptor subfamily 4 group A member 1 |
| NRAS | GTPase NRas (EC 3.6.5.2) (Transforming protein N-Ras) |
| NRBF2 | Nuclear receptor-binding factor 2 (NRBF-2) (Comodulator of PPAR and RXR) |
| NRG1 | Neuregulin 1 |
| NSDHL | NAD(P) dependent steroid dehydrogenase-like |
| NSF | Vesicle-fusing ATPase |
| NSFL1C | Alternative protein NSFL1C |
| NUP210 | Nucleoporin 210 |
| NUPR1 | Nuclear protein 1, transcriptional regulator |
| OLR1 | Oxidized low-density lipoprotein receptor 1 |
| OMA1 | OMA1 zinc metallopeptidase |
| OPA1 | OPA1 mitochondrial dynamin like GTPase |
| OPRD1 | Delta-type opioid receptor (D-OR-1) (DOR-1) |
| OPRM1 | Mu opioid receptor |
| ORAI1 | Calcium release-activated calcium channel protein 1 (Protein orai-1) (Transmembrane protein 142A) |
| ORMDL3 | ORM1-like protein 3 |
| OS9 | Endoplasmic reticulum lectin |
| OSBP | Oxysterol binding protein |
| OSBPL3 | Oxysterol binding protein like 3 |
| OSBPL8 | Oxysterol-binding protein |
| OXSR1 | Oxidative stress responsive kinase 1 |
| OXT | Oxytocin-neurophysin 1 (OT-NPI) [Cleaved into: Oxytocin (Ocytocin); Neurophysin 1] |
| OXTR | Oxytocin receptor (OT-R) |
| P3H1 | Prolyl 3-hydroxylase 1 |
| P3H4 | Prolyl 3-hydroxylase family member 4 (inactive) |
| P4HB | Prolyl 4-hydroxylase subunit beta |
| P4HTM | Prolyl 4-hydroxylase, transmembrane |
| PABPC1 | Poly(A) binding protein cytoplasmic 1 |
| PACS2 | Phosphofurin acidic cluster sorting protein 2 |
| PALS1 | Protein associated with LIN7 1, MAGUK p55 family member |
| PARG | Poly(ADP-ribose) glycohydrolase |
| PARK7 | Parkinsonism associated deglycase |
| PARP1 | Poly(ADP-ribose) polymerase 1 |
| PARP16 | Poly(ADP-ribose) polymerase family member 16 |
| PCK1 | Phosphoenolpyruvate carboxykinase, cytosolic [GTP] (PEPCK-C) (EC 4.1.1.32) (Serine-protein kinase PCK1) (EC 2.7.11.-) |
| PCNA | Proliferating cell nuclear antigen |
| PCSK6 | Proprotein convertase subtilisin/kexin type 6 |
| PCSK9 | Proprotein convertase subtilisin/kexin type 9 (Proprotein convertase 9) (Subtilisin/kexin-like protease PC9) |
| PDCD6 | Programmed cell death 6 |
| PDE5A | Phosphodiesterase 5A |
| PDHA1 | Pyruvate dehydrogenase E1 subunit alpha 1 |
| PDHB | Pyruvate dehydrogenase E1 component subunit beta (EC 1.2.4.1) |
| PDIA2 | Protein disulfide isomerase family A member 2 |
| PDIA3 | Protein disulfide isomerase family A member 3 |
| PDIA4 | Protein disulfide-isomerase (EC 5.3.4.1) |
| PDIA5 | Protein disulfide isomerase family A member 5 |
| PDIA6 | Protein disulfide isomerase family A member 6 |
| PDLIM1 | PDZ and LIM domain protein 1 (C-terminal LIM domain protein 1) (Elfin) (LIM domain protein CLP-36) |
| PDX1 | Pancreas/duodenum homeobox protein 1 (PDX-1) (Glucose-sensitive factor) (GSF) (Insulin promoter factor 1) (IPF-1) (Insulin upstream factor 1) (IUF-1) (Islet/duodenum homeobox-1) (IDX-1) (Somatostatin-transactivating factor 1) (STF-1) |
| PDZD8 | PDZ domain-containing protein 8 (Sarcoma antigen NY-SAR-84/NY-SAR-104) |
| PEF1 | Peflin (PEF protein with a long N-terminal hydrophobic domain) (Penta-EF hand domain-containing protein 1) |
| PEMT | Phosphatidylethanolamine N-methyltransferase |
| PEX11B | Peroxisomal biogenesis factor 11 beta |
| PGRMC1 | Membrane-associated progesterone receptor component 1 (mPR) (Dap1) (IZA) |
| PIEZO1 | Piezo-type mechanosensitive ion channel component |
| PIGA | Phosphatidylinositol glycan anchor biosynthesis class A |
| PIGB | Phosphatidylinositol glycan anchor biosynthesis class B |
| PIGBOS1 | Protein PIGBOS1 (PIGB opposite strand protein 1) |
| PIGC | Phosphatidylinositol N-acetylglucosaminyltransferase subunit C (Phosphatidylinositol-glycan biosynthesis class C protein) (PIG-C) |
| PIGH | Phosphatidylinositol N-acetylglucosaminyltransferase subunit H (Phosphatidylinositol-glycan biosynthesis class H protein) (PIG-H) |
| PIGK | GPI-anchor transamidase (Phosphatidylinositol-glycan biosynthesis class K protein) |
| PIGN | GPI ethanolamine phosphate transferase 1 (EC 2.-.-.-) |
| PIGS | Phosphatidylinositol glycan anchor biosynthesis class S |
| PIGT | Phosphatidylinositol glycan anchor biosynthesis class T |
| PIK3C3 | Phosphatidylinositol 3-kinase catalytic subunit type 3 |
| PIK3CA | Phosphatidylinositol 4,5-bisphosphate 3-kinase catalytic subunit alpha isoform (EC 2.7.1.153) |
| PIK3CG | Phosphatidylinositol-4,5-bisphosphate 3-kinase catalytic subunit gamma |
| PIK3R1 | Phosphatidylinositol 3-kinase regulatory subunit alpha (Phosphatidylinositol 3-kinase 85 kDa regulatory subunit alpha) |
| PIK3R2 | Phosphoinositide-3-kinase regulatory subunit 2 |
| PINK1 | Serine/threonine-protein kinase PINK1, mitochondrial (EC 2.7.11.1) (BRPK) (PTEN-induced putative kinase protein 1) |
| PITPNB | Phosphatidylinositol transfer protein beta |
| PITPNM1 | Phosphatidylinositol transfer protein membrane associated 1 |
| PKD1 | Polycystin 1, transient receptor potential channel interacting |
| PKD2 | Polycystin 2, transient receptor potential cation channel (cDNA FLJ50473, highly similar to Polycystin-2) |
| PKM | Pyruvate kinase M1/2 |
| PKP2 | Plakophilin-2 |
| PLA2G4A | Cytosolic phospholipase A2 alpha (EC 3.1.1.4) |
| PLA2G4C | Phospholipase A2, group IVC(Cytosolic, calcium-independent) |
| PLA2G6 | Phospholipase A2 group VI |
| PLCG1 | Phospholipase C gamma 1 |
| PLD3 | Phospholipase D family member 3 |
| PLEKHF2 | Pleckstrin homology domain-containing family F member 2 (PH domain-containing family F member 2) (Endoplasmic reticulum-associated apoptosis-involved protein containing PH and FYVE domains) (EAPF) (PH and FYVE domain-containing protein 2) (Phafin-2) (Phafin2) (Zinc finger FYVE domain-containing protein 18) |
| PLG | Plasminogen (EC 3.4.21.7) [Cleaved into: Plasmin heavy chain A; Activation peptide; Angiostatin; Plasmin heavy chain A, short form; Plasmin light chain B] |
| PLN | Cardiac phospholamban (PLB) |
| PLOD1 | Procollagen-lysine,2-oxoglutarate 5-dioxygenase 1 |
| PLOD2 | procollagen-lysine 5-dioxygenase (EC 1.14.11.4) |
| PLOD3 | Procollagen-lysine,2-oxoglutarate 5-dioxygenase 3 |
| PLP1 | Proteolipid protein 1 isoform 1 |
| PLPP3 | Phospholipid phosphatase 3 (EC 3.1.3.-) (EC 3.1.3.4) (Lipid phosphate phosphohydrolase 3) (PAP2-beta) (Phosphatidate phosphohydrolase type 2b) (Phosphatidic acid phosphatase 2b) (PAP-2b) (PAP2b) (Vascular endothelial growth factor and type I collagen-inducible protein) (VCIP) |
| PMAIP1 | Phorbol-12-myristate-13-acetate-induced protein 1 isoform 1 |
| PMEL | Alternative protein PMEL |
| PML | PML protein |
| PMM2 | Phosphomannomutase (EC 5.4.2.8) |
| PNKD | PNKD metallo-beta-lactamase domain containing |
| PNPLA6 | Mutant PNPLA6 protein |
| POFUT1 | GDP-fucose protein O-fucosyltransferase 1 (EC 2.4.1.221) (Peptide-O-fucosyltransferase 1) (O-FucT-1) |
| POGLUT2 | Protein O-glucosyltransferase 2 (EC 2.4.1.-) (Endoplasmic reticulum resident protein 58) (ER protein 58) (ERp58) (KDEL motif-containing protein 1) (Protein O-xylosyltransferase POGLUT2) (EC 2.4.2.-) |
| POMC | Pro-opiomelanocortin (POMC) (Corticotropin-lipotropin) [Cleaved into: NPP; Melanotropin gamma (Gamma-MSH); Potential peptide; Corticotropin (Adrenocorticotropic hormone) (ACTH); Melanocyte-stimulating hormone alpha (Alpha-MSH) (Melanotropin alpha); Corticotropin-like intermediary peptide (CLIP); Lipotropin beta (Beta-LPH); Lipotropin gamma (Gamma-LPH); Melanocyte-stimulating hormone beta (Beta-MSH) (Melanotropin beta); Beta-endorphin; Met-enkephalin] |
| POMP | Proteasome maturation protein |
| POMT1 | Protein O-mannosyl-transferase 1 (EC 2.4.1.109) (Dolichyl-phosphate-mannose--protein mannosyltransferase 1) |
| POMT2 | Dolichyl-phosphate-mannose--protein mannosyltransferase (EC 2.4.1.109) |
| PON1 | Paraoxonase (EC 3.1.1.2) |
| PON2 | Paraoxonase 2 |
| POP1 | POP1 homolog, ribonuclease P/MRP subunit |
| POR | Cytochrome p450 oxidoreductase |
| PPARA | Peroxisome proliferator-activated receptor alpha (PPAR-alpha) (Nuclear receptor subfamily 1 group C member 1) |
| PPARG | Peroxisome proliferator-activated receptor gamma (PPAR-gamma) (Nuclear receptor subfamily 1 group C member 3) |
| PPARGC1A | Peroxisome proliferator-activated receptor gamma coactivator 1-alpha |
| PPIA | Peptidyl-prolyl cis-trans isomerase (PPIase) (EC 5.2.1.8) |
| PPIB | Peptidyl-prolyl cis-trans isomerase (PPIase) (EC 5.2.1.8) |
| PPIF | Peptidyl-prolyl cis-trans isomerase (PPIase) (EC 5.2.1.8) |
| PPM1L | Protein phosphatase 1L (EC 3.1.3.16) (Protein phosphatase 1-like) (Protein phosphatase 2C isoform epsilon) (PP2C-epsilon) |
| PPP1CA | Protein phosphatase 1, catalytic subunit, alpha isoform |
| PPP1R15A | Protein phosphatase 1 regulatory subunit 15A (Growth arrest and DNA damage-inducible protein GADD34) (Myeloid differentiation primary response protein MyD116 homolog) |
| PPP1R15B | Protein phosphatase 1 regulatory subunit 15B |
| PPP2CB | Protein phosphatase 2 catalytic subunit beta |
| PPP3CA | Protein phosphatase 3 catalytic subunit alpha |
| PRDX1 | Peroxiredoxin 1 |
| PRDX2 | Peroxiredoxin 2, isoform CRA_d |
| PRDX3 | Epididymis secretory sperm binding protein (Peroxiredoxin 3, isoform CRA_b) |
| PRDX4 | Peroxiredoxin 4 |
| PRDX5 | Peroxiredoxin-5, mitochondrial (EC 1.11.1.24) (Alu corepressor 1) (Antioxidant enzyme B166) (AOEB166) (Liver tissue 2D-page spot 71B) (PLP) (Peroxiredoxin V) (Prx-V) (Peroxisomal antioxidant enzyme) (TPx type VI) (Thioredoxin peroxidase PMP20) (Thioredoxin-dependent peroxiredoxin 5) |
| PRDX6 | Peroxiredoxin-6 (EC 1.11.1.27) (1-Cys peroxiredoxin) (1-Cys PRX) (24 kDa protein) (Acidic calcium-independent phospholipase A2) (aiPLA2) (EC 3.1.1.4) (Antioxidant protein 2) (Glutathione-dependent peroxiredoxin) (Liver 2D page spot 40) (Lysophosphatidylcholine acyltransferase 5) (LPC acyltransferase 5) (LPCAT-5) (Lyso-PC acyltransferase 5) (EC 2.3.1.23) (Non-selenium glutathione peroxidase) (NSGPx) (Red blood cells page spot 12) |
| PREB | Prolactin regulatory element-binding protein (Mammalian guanine nucleotide exchange factor mSec12) |
| PREP | Prolyl endopeptidase |
| PRKAA1 | PRKAA1 protein (Protein kinase AMP-activated catalytic subunit alpha 1) (Protein kinase, AMP-activated, alpha 1 catalytic subunit, isoform CRA_b) |
| PRKAA2 | AMP-activated protein kinase alpha-2 subunit variant 2 |
| PRKAB1 | Protein kinase AMP-activated non-catalytic subunit beta 1 |
| PRKCA | Protein kinase C (EC 2.7.11.13) |
| PRKCD | Protein kinase C delta |
| PRKCQ | Protein kinase C theta type (EC 2.7.11.13) (nPKC-theta) |
| PRKCSH | Protein kinase C substrate 80K-H |
| PRKD1 | Protein kinase D1 |
| PRKDC | Protein kinase, DNA-activated, catalytic subunit |
| PRKN | Parkin RBR E3 ubiquitin protein ligase |
| PRKRA | Protein activator of interferon induced protein kinase EIF2AK2 |
| PRL | Prolactin (PRL) |
| PRNP | Major prion protein |
| PROC | Protein C, inactivator of coagulation factors Va and VIIIa |
| PROS1 | Protein S isoform 1 |
| PSEN1 | Presenilin 1 isoform 3 |
| PSEN2 | Presenilin-2 |
| PSENEN | Gamma-secretase subunit PEN-2 |
| PSMA7 | Proteasome 20S subunit alpha 7 |
| PSMC6 | Proteasome 26S subunit, ATPase 6 |
| PSMD2 | Proteasome 26S subunit ubiquitin receptor, non-ATPase 2 |
| PTEN | Mitochondrial PTENalpha |
| PTGIS | Alternative protein PTGIS |
| PTGS1 | Prostaglandin-endoperoxide synthase 1 |
| PTGS2 | Prostaglandin-endoperoxide synthase 2 |
| PTK2 | Protein tyrosine kinase 2 |
| PTP4A1 | Protein tyrosine phosphatase 4A1 |
| PTPA | Serine/threonine-protein phosphatase 2A activator (EC 5.2.1.8) (Phosphotyrosyl phosphatase activator) |
| PTPN1 | Tyrosine-protein phosphatase non-receptor type (EC 3.1.3.48) |
| PTPN11 | Protein tyrosine phosphatase-2 |
| PTPN2 | Protein tyrosine phosphatase non-receptor type 2 |
| PTPRC | Protein tyrosine phosphatase receptor type C |
| PURA | Purine rich element binding protein A |
| PXN | Paxillin |
| QDPR | Quinoid dihydropteridine reductase |
| QRICH1 | Alternative protein QRICH1 |
| RAB10 | Uncharacterized protein RAB10 |
| RAB18 | Ras-related protein Rab-18 |
| RAB1A | RAB1A, member RAS oncogene family |
| RAB1B | RAB1B protein |
| RAB2A | RAB2A, member RAS oncogene family |
| RAB3GAP1 | Rab3 GTPase-activating protein catalytic subunit |
| RAB6A | RAB6A, member RAS oncogene family |
| RAC1 | Rac family small GTPase 1 |
| RACK1 | Receptor for activated C kinase 1 |
| RAF1 | Raf-1 proto-oncogene, serine/threonine kinase |
| RAP1GDS1 | Rap1 GTPase-GDP dissociation stimulator 1 |
| RARA | Retinoic acid receptor alpha |
| RASGRF1 | Ras-specific guanine nucleotide-releasing factor 1 (Ras-GRF1) (Guanine nucleotide-releasing protein) (GNRP) (Ras-specific nucleotide exchange factor CDC25) |
| RASGRF2 | Ras protein specific guanine nucleotide releasing factor 2 |
| RCN1 | Reticulocalbin 1 |
| RCN2 | Alternative protein RCN2 |
| RCN3 | Reticulocalbin 3 |
| REEP1 | Receptor expression-enhancing protein |
| REEP4 | Receptor accessory protein 4 |
| REEP5 | Receptor expression-enhancing protein |
| RELA | Alternative protein RELA |
| RER1 | Protein RER1 |
| RET | Ret proto-oncogene |
| RETREG1 | Reticulophagy regulator 1 |
| RFT1 | Protein RFT1 homolog |
| RHBDD1 | Rhomboid domain containing 1 |
| RHBDD2 | Rhomboid domain-containing protein 2 |
| RHOA | Ras homolog family member A |
| RIC3 | Resistance to inhibitors of cholinesterase 3-like protein isoform 3 |
| RINT1 | RAD50-interacting protein 1 (RAD50 interactor 1) (HsRINT-1) (RINT-1) |
| RMRP | RMRP |
| RNF103 | E3 ubiquitin-protein ligase RNF103 (EC 2.3.2.27) (KF-1) (hKF-1) (RING finger protein 103) (RING-type E3 ubiquitin transferase RNF103) (Zinc finger protein 103 homolog) (Zfp-103) |
| RNF121 | Ring finger protein 121 |
| RNF13 | Ring finger protein 13 |
| RNF139 | Ring finger protein 139 |
| RNF175 | RING finger protein 175 |
| RNF183 | E3 ubiquitin-protein ligase RNF183 (EC 2.3.2.27) |
| RNF185 | E3 ubiquitin-protein ligase RNF185 (EC 2.3.2.27) (RING finger protein 185) |
| RNF186 | E3 ubiquitin-protein ligase RNF186 (EC 2.3.2.27) (RING finger protein 186) |
| RNF19B | E3 ubiquitin-protein ligase RNF19B (EC 2.3.2.31) (IBR domain-containing protein 3) (Natural killer lytic-associated molecule) (RING finger protein 19B) |
| RNF5 | E3 ubiquitin-protein ligase RNF (EC 2.3.2.27) (RING finger protein) |
| RNFT1 | Ring finger protein, transmembrane 1 |
| RNFT2 | Ring finger protein, transmembrane 2 |
| ROCK1 | Rho associated coiled-coil containing protein kinase 1 |
| RORA | RAR related orphan receptor A |
| RPA1 | Replication protein A1 |
| RPE65 | Mutant retinal pigment epithelium 65KDa protein |
| RPL10 | Ribosomal protein L10 |
| RPLP0 | 60S acidic ribosomal protein P0 |
| RPN1 | Dolichyl-diphosphooligosaccharide--protein glycosyltransferase subunit 1 (Dolichyl-diphosphooligosaccharide--protein glycosyltransferase 67 kDa subunit) (Ribophorin I) (RPN-I) (Ribophorin-1) |
| RPN2 | Dolichyl-diphosphooligosaccharide--protein glycosyltransferase subunit 2 (Dolichyl-diphosphooligosaccharide--protein glycosyltransferase 63 kDa subunit) (RIBIIR) (Ribophorin II) (RPN-II) (Ribophorin-2) |
| RPS27A | Ubiquitin-40S ribosomal protein S27a |
| RPS3 | Ribosomal protein S3 |
| RPS6 | 40S ribosomal protein S6 |
| RPS6KA3 | Ribosomal protein S6 kinase A3 |
| RPTOR | Regulatory associated protein of MTOR complex 1 |
| RRBP1 | Ribosome binding protein 1 |
| RSAD2 | Radical S-adenosyl methionine domain containing 2 |
| RTN1 | Reticulon 1 isoform A |
| RTN2 | Truncated RTN2-A2 |
| RTN3 | Truncated RTN3-A3 |
| RTN4 | Uncharacterized protein RTN4 |
| RUVBL2 | RuvB like AAA ATPase 2 |
| RYR1 | Ryanodine receptor type1 |
| RYR2 | Alternative protein RYR2 |
| RYR3 | Ryanodine receptor 3 |
| S100A1 | S100 calcium binding protein A1 |
| S100A9 | Protein S100-A9 (Calgranulin-B) (Calprotectin L1H subunit) (Leukocyte L1 complex heavy chain) (Migration inhibitory factor-related protein 14) (MRP-14) (p14) (S100 calcium-binding protein A9) |
| SACM1L | SAC1 like phosphatidylinositide phosphatase |
| SAMD8 | Sterile alpha motif domain containing 8 |
| SAR1A | Secretion associated Ras related GTPase 1A |
| SAR1B | Secretion associated Ras related GTPase 1B |
| SCAMP5 | Secretory carrier-associated membrane protein 5 (Secretory carrier membrane protein 5) (hSCAMP5) |
| SCAP | SREBF chaperone |
| SCAPER | S-phase cyclin A associated protein in the ER |
| SCARA3 | Scavenger receptor class A member 3 (Cellular stress response gene protein) |
| SCD | Stearoyl-CoA desaturase (hSCD1) (EC 1.14.19.1) (Acyl-CoA desaturase) (Delta(9)-desaturase) (Delta-9 desaturase) (Fatty acid desaturase) |
| SCFD1 | Sec1 family domain containing 1 |
| SCN10A | Sodium channel protein |
| SCN1A | Sodium channel protein |
| SCN5A | Sodium channel protein |
| SCP2 | Sterol carrier protein 2 (SCP-2) (Acetyl-CoA C-myristoyltransferase) (EC 2.3.1.155) (Non-specific lipid-transfer protein) (NSL-TP) (Propanoyl-CoA C-acyltransferase) (EC 2.3.1.176) (SCP-2/3-oxoacyl-CoA thiolase) (SCP-2/thiolase) (EC 2.3.1.16) (SCP-chi) (SCPX) (Sterol carrier protein X) (SCP-X) |
| SCYL1 | SCY1 like pseudokinase 1 |
| SDF2L1 | Stromal cell-derived factor 2-like protein 1 (SDF2-like protein 1) (PWP1-interacting protein 8) |
| SDHA | succinate dehydrogenase (EC 1.3.5.1) |
| SDHB | Succinate dehydrogenase complex, subunit B, iron sulfur (Ip) (EC 1.3.5.1) |
| SEC11A | SEC11 homolog A, signal peptidase complex subunit |
| SEC13 | SEC13 homolog, nuclear pore and COPII coat complex component |
| SEC16A | SEC16 homolog A, endoplasmic reticulum export factor |
| SEC22A | SEC22 homolog A, vesicle trafficking protein |
| SEC22B | SEC22 homolog B, vesicle trafficking protein |
| SEC22C | SEC22 homolog C, vesicle trafficking protein |
| SEC23A | Protein transport protein SEC23 |
| SEC23B | Protein transport protein SEC23 |
| SEC23IP | SEC23-interacting protein (p125) |
| SEC24A | Protein transport protein Sec24A (SEC24-related protein A) |
| SEC24B | Protein transport protein Sec24B (SEC24-related protein B) |
| SEC24C | SEC24 homolog C, COPII coat complex component |
| SEC24D | SEC24 homolog D, COPII coat complex component |
| SEC31A | Protein transport protein Sec31A |
| SEC31B | SEC31 homolog B, COPII coat complex component |
| SEC61A1 | SEC61 translocon subunit alpha 1 (cDNA FLJ59739, highly similar to Protein transport protein Sec61 subunit alpha isoform 1) |
| SEC61B | Protein transport protein Sec61 subunit beta |
| SEC61G | Protein transport protein Sec61 subunit gamma |
| SEC62 | SEC62 homolog, preprotein translocation factor |
| SEC63 | SEC63 homolog, protein translocation regulator |
| SEL1L | Sel-1-like protein |
| SEL1L2 | SEL1L2 adaptor subunit of ERAD E3 ligase |
| SELENOF | Selenoprotein F |
| SELENOK | Selenoprotein K |
| SELENON | Selenoprotein N |
| SELENOS | Selenoprotein S |
| SELENOT | Selenoprotein T |
| SELP | Selectin P isoform 3 |
| SERINC3 | Alternative protein SERINC3 |
| SERP1 | Stress-associated endoplasmic reticulum protein |
| SERP2 | Stress-associated endoplasmic reticulum protein |
| SERPINA1 | Alpha-1-antitrypsin (Serpin family A member 1) |
| SERPINA2 | Alpha-1-antitrypsin-related protein |
| SERPINC1 | Antithrombin-III (Serpin C1) |
| SERPINE1 | Plasminogen activator inhibitor 1 (PAI) (PAI-1) (Endothelial plasminogen activator inhibitor) (Serpin E1) |
| SERPINH1 | Serpin family H member 1 |
| SERPINI1 | Serpin family I member 1 |
| SESN2 | Sestrin-2 (EC 1.11.1.-) (Hypoxia-induced gene) |
| SET | SET nuclear proto-oncogene |
| SETD2 | [histone H3]-lysine(36) N-trimethyltransferase (EC 2.1.1.359) (SET domain-containing protein 2) |
| SFTPC | Pulmonary surfactant-associated protein C |
| SGF29 | SAGA complex associated factor 29 |
| SGK1 | Sgk1 variant i3 |
| SGPP1 | Sphingosine-1-phosphate phosphatase 1 (SPPase1) (Spp1) (hSPP1) (hSPPase1) (EC 3.1.3.-) (Sphingosine-1-phosphatase 1) (Sphingosine-1-phosphate phosphohydrolase 1) (SPP-1) |
| SGPP2 | Sphingosine-1-phosphate phosphatase 2 (SPPase2) (Spp2) (hSPP2) (EC 3.1.3.-) (Sphingosine-1-phosphatase 2) |
| SGSH | N-sulfoglucosamine sulfohydrolase |
| SGTA | Small glutamine rich tetratricopeptide repeat co-chaperone alpha |
| SGTB | Small glutamine rich tetratricopeptide repeat co-chaperone beta |
| SHC1 | SHC adaptor protein 1 |
| SHH | Sonic hedgehog signaling molecule |
| SHISA5 | Shisa family member 5 |
| SI | Sucrase-isomaltase |
| SIGMAR1 | Sigma non-opioid intracellular receptor 1 (Sigma 1-type opioid receptor) |
| SIL1 | Endoplasmic reticulum chaperone |
| SIRT1 | Sirtuin 1 |
| SIRT2 | Sirtuin 2 |
| SIRT3 | Sirtuin 3 |
| SLC1A1 | Amino acid transporter |
| SLC25A1 | Plasma membrane citrate carrier |
| SLC27A2 | Long-chain fatty acid transport protein 2 (Arachidonate--CoA ligase) (EC 6.2.1.15) (Fatty acid transport protein 2) (FATP-2) (Fatty-acid-coenzyme A ligase, very long-chain 1) (Long-chain-fatty-acid--CoA ligase) (EC 6.2.1.3) (Phytanate--CoA ligase) (EC 6.2.1.24) (Solute carrier family 27 member 2) (THCA-CoA ligase) (EC 6.2.1.7) (Very long-chain acyl-CoA synthetase) (VLACS) (VLCS) (EC 6.2.1.-) (Very long-chain-fatty-acid-CoA ligase) |
| SLC2A1 | Solute carrier family 2 member 1 |
| SLC2A4 | Solute carrier family 2 member 4 |
| SLC35B1 | Glucose-6-phosphate exchanger SLC37A1 (Glycerol-3-phosphate permease) (G-3-P permease) (Solute carrier family 37 member 1) |
| SLC37A1 | Solute carrier family 37 member 1 |
| SLC37A4 | Solute carrier family 37 member 4 |
| SLC39A14 | Solute carrier family 39 member 14 |
| SLC39A7 | Solute carrier family 39 member 7 |
| SLC4A1 | Solute carrier family 4 member 1 (Diego blood group) |
| SLC6A1 | Solute carrier family 6 member 1 |
| SLC6A4 | Solute carrier family 6 member 4 |
| SLC8A1 | Solute carrier family 8 member A1 |
| SLC8A3 | Solute carrier family 8 member A3 |
| SLC9A1 | Solute carrier family 9 member A1 |
| SLN | Sarcolipin |
| SMN1 | Survival motor neuron protein |
| SMPD1 | Sphingomyelin phosphodiesterase 1 |
| SMPD2 | Sphingomyelin phosphodiesterase 2 |
| SMPD4 | Sphingomyelin phosphodiesterase 4 |
| SNAP25 | Synaptosomal-associated protein 25 (SNAP-25) |
| SNCA | Alpha-synuclein |
| SNTA1 | SNTA1 protein |
| SOAT1 | Sterol O-acyltransferase 1 |
| SOAT2 | Sterol O-acyltransferase 2 |
| SOD1 | Superoxide dismutase 1 |
| SOD2 | Superoxide dismutase (EC 1.15.1.1) |
| SOD3 | Superoxide dismutase 3 |
| SORL1 | Sortilin related receptor 1 |
| SORT1 | Sortilin 1 |
| SP1 | Specificity protein 1 |
| SPAG5 | Sperm associated antigen 5 |
| SPAST | Spastin |
| SPCS2 | Signal peptidase complex subunit 2 |
| SPP1 | Secreted phosphoprotein 1 |
| SPTLC1 | Alternative protein SPTLC1 |
| SQSTM1 | Sequestosome 1 |
| SRC | Tyrosine kinase pp60c-src |
| SREBF1 | Sterol regulatory element binding transcription factor 1 |
| SREBF2 | Sterol regulatory element binding transcription factor 2 |
| SRI | Sorcin (22 kDa protein) (CP-22) (CP22) (V19) |
| SRL | Alternative protein SRL |
| SRP14 | Signal recognition particle 14 |
| SRP54 | Signal recognition particle 54 |
| SRP68 | Signal recognition particle 68 |
| SRP72 | Signal recognition particle 72 |
| SRPRA | Signal recognition particle receptor subunit alpha (SR-alpha) (Docking protein alpha) (DP-alpha) |
| SRPRB | Signal recognition particle receptor subunit beta |
| SRPX | Sushi repeat-containing protein SRPX |
| SSR1 | Translocon-associated protein subunit alpha (TRAP-alpha) (Signal sequence receptor subunit alpha) |
| SSR2 | Signal sequence receptor subunit 2 |
| SSR3 | Translocon-associated protein subunit gamma (Signal sequence receptor subunit gamma) |
| SSR4 | Translocon-associated protein subunit delta (Signal sequence receptor subunit delta) |
| STARD3 | StAR related lipid transfer domain containing 3 |
| STARD3NL | STARD3 N-terminal like |
| STARD5 | StAR-related lipid transfer protein 5 (START domain-containing protein 5) (StARD5) |
| STAT1 | Signal transducer and activator of transcription 1-alpha/beta (Transcription factor ISGF-3 components p91/p84) |
| STAT3 | Signal transducer and activator of transcription 3 |
| STAU1 | Staufen double-stranded RNA binding protein 1 |
| STC2 | Stanniocalcin-2 (STC-2) (Stanniocalcin-related protein) (STC-related protein) (STCRP) |
| STEEP1 | STING ER exit protein (STEEP) |
| STIM1 | Stromal interaction molecule 1 |
| STIM2 | Stromal interaction molecule 2 |
| STING1 | Stimulator of interferon genes protein |
| STIP1 | Stress induced phosphoprotein 1 |
| STK25 | Serine/threonine kinase 25 |
| STK39 | non-specific serine/threonine protein kinase (EC 2.7.11.1) |
| STT3A | dolichyl-diphosphooligosaccharide--protein glycotransferase (EC 2.4.99.18) |
| STT3B | Dolichyl-diphosphooligosaccharide--protein glycosyltransferase subunit STT3B (Oligosaccharyl transferase subunit STT3B) (STT3-B) (EC 2.4.99.18) (Source of immunodominant MHC-associated peptides homolog) |
| STUB1 | STIP1 homology and U-box containing protein 1 |
| STX17 | Syntaxin-17 |
| STX18 | Syntaxin 18 |
| STX5 | Syntaxin 5 |
| STXBP1 | Syntaxin binding protein 1 |
| SUMF2 | Sulfatase modifying factor 2 isoform 2 |
| SURF4 | Surfeit locus protein 4 |
| SVIP | Small VCP/p97-interacting protein |
| SYNCRIP | Synaptotagmin binding cytoplasmic RNA interacting protein |
| SYT2 | Synaptotagmin |
| SYVN1 | Synoviolin 1 |
| TANGO2 | Transport and golgi organization 2 homolog |
| TAOK3 | TAO kinase 3 |
| TAP1 | Transporter 1 ATP-binding cassette sub-family B isoform 2 |
| TAP2 | Antigen peptide transporter 2 (APT2) (EC 7.4.2.14) (ATP-binding cassette sub-family B member 3) (Peptide supply factor 2) (Peptide transporter PSF2) (PSF-2) (Peptide transporter TAP2) (Peptide transporter involved in antigen processing 2) (Really interesting new gene 11 protein) (RING11) |
| TAPBP | TAP binding protein (Tapasin) |
| TAPBPL | Tapasin-related protein (TAPASIN-R) (TAP-binding protein-like) (TAP-binding protein-related protein) (TAPBP-R) (Tapasin-like) |
| TARDBP | TAR DNA binding protein |
| TBL2 | Transducin beta like 2 |
| TBXAS1 | Thromboxane A synthase 1 |
| TECR | Trans-2,3-enoyl-CoA reductase |
| TECRL | Trans-2,3-enoyl-CoA reductase like |
| TERT | Telomerase catalytic subunit |
| TESPA1 | Thymocyte expressed, positive selection associated 1 |
| TEX2 | Testis expressed 2 |
| TEX264 | Testis expressed 264, ER-phagy receptor |
| TF | Transferrin |
| TFEB | Transcription factor EB |
| TFG | Trafficking from ER to golgi regulator |
| TFRC | Transferrin receptor protein 1 |
| TG | Thyroglobulin |
| TGFA | Transforming growth factor alpha |
| TGFB1 | Transforming growth factor, beta 1 |
| TGFBR1 | Transforming growth factor beta receptor 1 |
| TGM2 | Transglutaminase 2 |
| TH | Tyrosine hydroxylase |
| THBS1 | Thrombospondin 1 |
| THBS4 | Thrombospondin 4 |
| TIA1 | Alternative protein TIA1 |
| TIAL1 | Nucleolysin TIAR (TIA-1-related protein) |
| TJP1 | Tight junction protein 1 |
| TLR2 | Toll-like receptor |
| TLR3 | Toll like receptor 3 |
| TLR4 | Toll like receptor 4 |
| TLR7 | Toll-like receptor 7 |
| TLR9 | Alternative protein TLR9 |
| TM7SF2 | Transmembrane 7 superfamily member 2 |
| TMBIM6 | Transmembrane BAX inhibitor motif containing 6 |
| TMCC1 | Transmembrane and coiled-coil domain family 1 |
| TMCO1 | Alternative protein TMCO1 |
| TMED1 | Transmembrane p24 trafficking protein 1 |
| TMED10 | Transmembrane p24 trafficking protein 10 |
| TMED2 | Transmembrane p24 trafficking protein 2 |
| TMED4 | Transmembrane p24 trafficking protein 4 |
| TMED7 | Transmembrane p24 trafficking protein 7 |
| TMED9 | Transmembrane emp24 domain-containing protein 9 (GMP25) (Glycoprotein 25L2) (p24 family protein alpha-2) (p24alpha2) (p25) |
| TMEM117 | Transmembrane protein 117 |
| TMEM129 | Transmembrane protein 129, E3 ubiquitin ligase |
| TMEM199 | Transmembrane protein 199 |
| TMEM208 | Transmembrane protein 208 |
| TMEM214 | Transmembrane protein 214 |
| TMEM259 | Transmembrane protein 259 |
| TMEM33 | Transmembrane protein 33 |
| TMEM43 | Transmembrane protein 43 |
| TMEM67 | Transmembrane protein 67 |
| TMTC3 | Alternative protein TMTC3 |
| TMTC4 | Protein O-mannosyl-transferase TMTC4 (EC 2.4.1.109) (Transmembrane and TPR repeat-containing protein 4) |
| TMUB1 | Transmembrane and ubiquitin like domain containing 1 |
| TMUB2 | Transmembrane and ubiquitin like domain containing 2 |
| TMX1 | Thioredoxin related transmembrane protein 1 |
| TMX2 | Thioredoxin related transmembrane protein 2 |
| TMX3 | Thioredoxin related transmembrane protein 3 |
| TNF | Tumor necrosis factor (TNF-a) (Cachectin) (TNF-alpha) (Tumor necrosis factor ligand superfamily member 2) [Cleaved into: Intracellular domain 1 (ICD1); Intracellular domain 2 (ICD2); C-domain 1; C-domain 2; Tumor necrosis factor, soluble form] |
| TNFRSF10B | TNF receptor superfamily member 10b |
| TNFRSF1A | TNF receptor superfamily member 1A |
| TNFSF10 | TNF superfamily member 10 |
| TOR1A | Torsin family 1 member A |
| TOR1AIP1 | Torsin 1A interacting protein 1 |
| TOR1AIP2 | Torsin 1A interacting protein 2 |
| TOR1B | Torsin family 1 member B |
| TP53 | Cellular tumor antigen p53 |
| TPM1 | Tropomyosin 1 |
| TRA | M1-specific T cell receptor alpha chain (TR alpha chain TRAV27*01J42*01C*01) |
| TRAF2 | TNF receptor-associated factor 2 (EC 2.3.2.27) (E3 ubiquitin-protein ligase TRAF2) (RING-type E3 ubiquitin transferase TRAF2) (Tumor necrosis factor type 2 receptor-associated protein 3) |
| TRAF6 | TNF receptor-associated factor 6 (EC 2.3.2.27) (E3 ubiquitin-protein ligase TRAF6) (Interleukin-1 signal transducer) (RING finger protein 85) (RING-type E3 ubiquitin transferase TRAF6) |
| TRAM1 | Translocation associated membrane protein 1 |
| TRAM2 | Translocating chain-associated membrane protein 2 |
| TRAP1 | TNF receptor associated protein 1 |
| TRAPPC11 | Trafficking protein particle complex subunit 11 |
| TRAPPC12 | Trafficking protein particle complex subunit 12 |
| TRAPPC2 | Trafficking protein particle complex subunit 2 |
| TRAPPC3 | Trafficking protein particle complex subunit |
| TRAPPC4 | Trafficking protein particle complex subunit |
| TRAPPC5 | Trafficking protein particle complex subunit 5 |
| TRAPPC9 | Trafficking protein particle complex subunit 9 |
| TRDN | Triadin |
| TREM2 | Triggering receptor expressed on myeloid cells 2 (TREM-2) (Triggering receptor expressed on monocytes 2) |
| TRIB3 | Tribbles pseudokinase 3 |
| TRIM13 | Alternative protein TRIM13 |
| TRIM25 | Tripartite motif containing 25 |
| TRIP11 | Thyroid hormone receptor interactor 11 |
| TRPA1 | Transient receptor potential cation channel subfamily A member 1 |
| TRPC1 | Short transient receptor potential channel 1 |
| TRPM2 | Transient receptor potential cation channel subfamily M member 2 |
| TRPM4 | Transient receptor potential cation channel subfamily M member 4 |
| TRPM8 | Transient receptor melastatin 8 variant 11 |
| TRPV1 | Transient receptor potential cation channel subfamily V member 1 |
| TRPV4 | Transient receptor potential cation channel subfamily V member 4 |
| TSC1 | TSC complex subunit 1 |
| TSPO | Translocator protein |
| TTC23L | Tetratricopeptide repeat domain 23 like |
| TTF2 | Transcription termination factor 2 |
| TTN | Transthyretin |
| TTR | TTR protein |
| TUSC3 | Tumor suppressor candidate 3 |
| TXN | Mitochondrial thioredoxin |
| TXNDC12 | Thioredoxin domain containing 12 |
| TXNDC5 | protein disulfide-isomerase (EC 5.3.4.1) |
| TXNIP | Alternative protein TXNIP |
| TXNRD1 | Thioredoxin reductase 1 |
| TYR | Tyrosinase |
| UBA5 | Ubiquitin like modifier activating enzyme 5 |
| UBA52 | Ubiquitin A-52 residue ribosomal protein fusion product 1 |
| UBAC2 | UBA domain containing 2 |
| UBB | Ubiquitin B |
| UBC | Ubiquitin C |
| UBE2D1 | Ubiquitin conjugating enzyme E2 D1 |
| UBE2D2 | Ubiquitin conjugating enzyme E2 D2 |
| UBE2D3 | Ubiquitin conjugating enzyme E2 D3 |
| UBE2G2 | Ubiquitin conjugating enzyme E2 G2 |
| UBE2J1 | Ubiquitin-conjugating enzyme E2 J1 (EC 2.3.2.23) (E2 ubiquitin-conjugating enzyme J1) (Non-canonical ubiquitin-conjugating enzyme 1) (NCUBE-1) (Yeast ubiquitin-conjugating enzyme UBC6 homolog E) (HsUBC6e) |
| UBE2J2 | Ubiquitin conjugating enzyme E2 J2 |
| UBE2K | Ubiquitin conjugating enzyme E2 K |
| UBE2N | Ubiquitin conjugating enzyme E2 N |
| UBE4A | Alternative protein UBE4A |
| UBE4B | Ubiquitination factor E4B |
| UBL4A | Ubiquitin-like protein 4A |
| UBQLN1 | Ubiquilin 1 |
| UBQLN2 | Ubiquilin-2 (Chap1) (DSK2 homolog) (Protein linking IAP with cytoskeleton 2) (PLIC-2) (hPLIC-2) (Ubiquitin-like product Chap1/Dsk2) |
| UBQLN4 | Ubiquilin 4, isoform CRA_a |
| UBXN1 | UBX domain protein 1 |
| UBXN10 | UBXN10 protein |
| UBXN2B | UBX domain protein 2B |
| UBXN4 | UBX domain protein 4 |
| UBXN6 | UBX domain protein 6 |
| UBXN8 | UBX domain protein 8 |
| UCHL1 | Ubiquitin C-terminal hydrolase L1 |
| UCP2 | Uncoupling protein 2 |
| UFC1 | Ubiquitin-fold modifier-conjugating enzyme 1 (Ufm1-conjugating enzyme 1) |
| UFD1 | Ubiquitin recognition factor in ER associated degradation 1 |
| UFL1 | E3 UFM1-protein ligase 1 (EC 2.3.2.-) (E3 UFM1-protein transferase 1) (Multiple alpha-helix protein located at ER) (Novel LZAP-binding protein) (Regulator of C53/LZAP and DDRGK1) |
| UFM1 | Ubiquitin-fold modifier 1 |
| UGGT1 | UDP-glucose glycoprotein glucosyltransferase 1 |
| UGGT2 | UDP-glucose glycoprotein glucosyltransferase 2 |
| UGT1A | Bilirubin UDP-glucuronosyltranserase |
| UGT1A1 | Bilirubin UDP-glucronosyltrasferase 1-1 |
| UGT1A10 | UDP-glucuronosyltransferase (EC 2.4.1.17) |
| UGT1A3 | UDP-glucuronosyltransferase 1A3 |
| UGT1A4 | UDP-glucuronosyltransferase 1A4 |
| UGT1A5 | UDP-glucuronosyltransferase 1A5 (UGT1A5) (EC 2.4.1.17) (UDP-glucuronosyltransferase 1-5) (UDPGT 1-5) (UGT1*5) (UGT1-05) (UGT1.5) (UDP-glucuronosyltransferase 1-E) (UGT-1E) (UGT1E) |
| UGT1A6 | UDP-glucuronosyltransferase 1-6 (UDPGT 1-6) (UGT1*6) (UGT1-06) (UGT1.6) (EC 2.4.1.17) (Phenol-metabolizing UDP-glucuronosyltransferase) (UDP-glucuronosyltransferase 1-F) (UGT-1F) (UGT1F) (UDP-glucuronosyltransferase 1A6) |
| UGT1A7 | UDP glucuronosyltransferase family 1 member A7 |
| UGT1A8 | UDP-glucuronosyltransferase 1A8 |
| UGT1A9 | UDP-glucuronosyltransferase 1A9 |
| ULBP1 | UL16-binding protein 1 (ALCAN-beta) (NKG2D ligand 1) (N2DL-1) (NKG2DL1) (Retinoic acid early transcript 1I) |
| UMOD | Uromodulin (Tamm-Horsfall urinary glycoprotein) (THP) [Cleaved into: Uromodulin, secreted form] |
| UNC93B1 | Unc-93 homolog B1, TLR signaling regulator |
| UQCRFS1 | Cytochrome b-c1 complex subunit Rieske, mitochondrial (EC 7.1.1.8) (Complex III subunit 5) (Cytochrome b-c1 complex subunit 5) (Rieske iron-sulfur protein) (RISP) (Rieske protein UQCRFS1) (Ubiquinol-cytochrome c reductase iron-sulfur subunit) [Cleaved into: Cytochrome b-c1 complex subunit 9 (Su9) (Subunit 9) (8 kDa subunit 9) (Complex III subunit IX) (Cytochrome b-c1 complex subunit 11) (UQCRFS1 mitochondrial targeting sequence) (UQCRFS1 MTS) (Ubiquinol-cytochrome c reductase 8 kDa protein)] |
| USE1 | Vesicle transport protein USE1 (USE1-like protein) |
| USO1 | Alternative protein USO1 |
| USP13 | Ubiquitin specific peptidase 13 |
| USP14 | Ubiquitin carboxyl-terminal hydrolase (EC 3.4.19.12) |
| USP19 | Ubiquitin specific peptidase 19 |
| USP25 | Ubiquitin specific peptidase 25 |
| USP9X | Ubiquitin specific peptidase 9 X-linked |
| UVRAG | UV radiation resistance associated |
| VAMP1 | Vesicle associated membrane protein 1 |
| VAMP7 | Vesicle associated membrane protein 7 |
| VAPA | VAMP associated protein A |
| VAPB | VAMP associated protein B and C |
| VCAM1 | Vascular cell adhesion molecule 1 |
| VCL | Vinculin (Metavinculin) |
| VCP | Valosin-containing protein |
| VCPIP1 | Deubiquitinating protein VCPIP1 (EC 3.4.19.12) (Valosin-containing protein p97/p47 complex-interacting protein 1) (Valosin-containing protein p97/p47 complex-interacting protein p135) (VCP/p47 complex-interacting 135-kDa protein) |
| VDAC1 | Voltage dependent anion channel 1 |
| VDR | Vitamin D receptor |
| VEGFA | Vascular endothelial growth factor A |
| VHL | von-Hippel Lindau tumor supressor isoform 1 |
| VIM | Vimentin |
| VKORC1 | vitamin-K-epoxide reductase (warfarin-sensitive) (EC 1.17.4.4) |
| VKORC1L1 | Vitamin K epoxide reductase complex subunit 1-like protein 1 (VKORC1-like protein 1) (EC 1.17.4.4) |
| VMA21 | Vacuolar ATPase assembly integral membrane protein VMA21 (Myopathy with excessive autophagy protein) |
| VMP1 | Vacuole membrane protein 1 |
| VPS33A | Alternative protein VPS33A |
| VRK2 | VRK serine/threonine kinase 2 |
| VWF | von Willebrand factor (vWF) |
| WDR83OS | PAT complex subunit Asterix |
| WFS1 | Wolframin ER transmembrane glycoprotein |
| WWOX | WW domain containing oxidoreductase |
| XBP1 | X-box binding protein 1 |
| XDH | Uncharacterized protein XDH |
| XIAP | E3 ubiquitin-protein ligase XIAP |
| YBX1 | Y-box binding protein 1 |
| YIF1A | Protein YIF1 |
| YIF1B | Protein YIF1B (YIP1-interacting factor homolog B) |
| YIPF5 | Yip1 domain family member 5 |
| YKT6 | YKT6 v-SNARE homolog |
| YOD1 | Ubiquitin thioesterase OTU1 (EC 3.4.19.12) (DUBA-8) (HIV-1-induced protease 7) (HIN-7) (HsHIN7) (OTU domain-containing protein 2) |
| YTHDF2 | YTH domain-containing family protein |
| YWHAE | 14-3-3 protein epsilon (14-3-3E) |
| YWHAZ | Tyrosine 3-monooxygenase/tryptophan 5-monooxygenase activation protein zeta (cDNA FLJ50142, highly similar to 14-3-3 protein zeta/delta (Protein kinase Cinhibitor protein 1)) |
| YY1 | YY1 transcription factor |
| ZC3H12A | Zinc finger CCCH-type containing 12A |
| ZDHHC4 | Zinc finger DHHC-type palmitoyltransferase 4 |
| ZDHHC6 | Palmitoyltransferase (EC 2.3.1.225) |
| ZFAND2B | Zinc finger AN1-type containing 2B |
| ZFYVE1 | Zinc finger FYVE-type containing 1 |
| ZFYVE27 | Protrudin (Zinc finger FYVE domain-containing protein 27) |
| ZMPSTE24 | Zinc metallopeptidase STE24 |
| ZW10 | Centromere/kinetochore protein zw10 homolog |

**Supplementary TABLE 3** The hub ERSRGs and their differential expression characteristics.

| id | logFC | AveExpr | *P*.Value | adj.*P*.Val |
| --- | --- | --- | --- | --- |
| CDKN1B | -0.597350768 | 8.650814809 | 6.53E-24 | 1.02E-20 |
| FN1 | 1.83537274 | 7.087288396 | 6.70E-22 | 2.72E-19 |
| NR3C2 | -0.616342865 | 6.605419608 | 7.99E-21 | 2.33E-18 |
| MMP2 | 0.979698567 | 5.970741384 | 8.35E-20 | 1.95E-17 |
| MAN2B1 | 0.61638407 | 6.167138028 | 2.30E-19 | 4.77E-17 |
| LPL | -1.272701338 | 6.534389749 | 2.92E-19 | 5.73E-17 |
| EXT1 | 0.516239047 | 7.445461036 | 3.91E-19 | 7.31E-17 |
| MARCKS | 0.985139185 | 6.225747827 | 1.06E-17 | 1.26E-15 |
| PDLIM1 | 0.563877509 | 9.436537609 | 2.36E-17 | 2.57E-15 |
| FOS | -1.788189412 | 6.500541538 | 4.11E-17 | 4.27E-15 |
| PLA2G4A | 0.799559045 | 4.187297123 | 2.75E-16 | 2.19E-14 |
| JUN | -0.658166537 | 6.176530442 | 6.17E-16 | 4.24E-14 |
| TPM1 | 0.603559054 | 8.520333024 | 1.21E-15 | 7.59E-14 |
| DHCR24 | 0.636508164 | 6.423551346 | 2.52E-15 | 1.45E-13 |
| CR1 | -0.792961269 | 7.418621879 | 4.15E-15 | 2.21E-13 |
| COL1A1 | 0.741479105 | 4.298376333 | 6.98E-15 | 3.49E-13 |
| CD36 | 0.810221868 | 4.199710841 | 2.70E-14 | 1.12E-12 |
| EZH2 | 0.55058877 | 3.784766148 | 2.99E-14 | 1.23E-12 |
| COMP | 1.107988184 | 4.086707169 | 3.26E-14 | 1.31E-12 |
| FBN1 | 0.671410737 | 5.702246006 | 4.16E-14 | 1.63E-12 |
| IGF1 | -1.337647123 | 7.097790556 | 4.87E-14 | 1.86E-12 |
| CAV1 | 0.688644338 | 6.18957095 | 1.42E-13 | 4.70E-12 |
| FBXO17 | -0.561878065 | 7.078060637 | 2.06E-13 | 6.57E-12 |
| KPNA2 | 0.579549902 | 8.053542155 | 4.46E-13 | 1.35E-11 |
| ATF3 | -1.071843326 | 6.209591914 | 1.02E-12 | 2.80E-11 |
| CLU | 0.977821989 | 6.799113071 | 1.09E-12 | 2.92E-11 |
| CYBB | 0.836879142 | 6.073973166 | 1.77E-12 | 4.47E-11 |
| NQO1 | -0.506937731 | 9.113772875 | 4.15E-12 | 9.63E-11 |
| EGR1 | -1.158027741 | 7.667739727 | 5.87E-12 | 1.30E-10 |
| NR4A1 | -0.615423417 | 6.020449141 | 2.25E-11 | 4.24E-10 |
| VEGFA | -0.505837183 | 8.170862995 | 4.89E-11 | 8.43E-10 |
| ZDHHC6 | -0.580803285 | 11.34540347 | 5.94E-11 | 9.96E-10 |
| NUPR1 | -0.560670456 | 7.200530115 | 8.22E-11 | 1.30E-09 |
| F3 | -0.634725281 | 8.406095329 | 1.64E-10 | 2.36E-09 |
| FCGR2B | 0.769351796 | 4.425836704 | 2.79E-10 | 3.76E-09 |
| UCP2 | 0.687404602 | 6.658431919 | 2.85E-10 | 3.84E-09 |
| TLR2 | 0.529373469 | 5.133590919 | 1.93E-09 | 2.16E-08 |
| PLN | 0.614798053 | 5.525285286 | 7.35E-09 | 7.09E-08 |
| COL4A1 | 0.556521101 | 7.942093312 | 1.97E-08 | 1.70E-07 |
| ELOVL4 | -0.536682378 | 5.615433051 | 2.38E-08 | 2.03E-07 |
| FKBP5 | -0.555989481 | 6.950876531 | 3.65E-08 | 3.00E-07 |
| AGR2 | 0.759556925 | 3.662661547 | 1.49E-07 | 1.06E-06 |
| CCL2 | 0.827601847 | 8.229597933 | 4.25E-07 | 2.71E-06 |
| DSP | 0.569576275 | 7.335204356 | 7.73E-07 | 4.70E-06 |
| GDF15 | -0.624942763 | 5.25765972 | 8.49E-07 | 5.11E-06 |
| PTPRC | 0.546647662 | 5.955357939 | 1.40E-06 | 7.99E-06 |
| RSAD2 | -0.513438068 | 5.961591401 | 1.32E-05 | 5.94E-05 |
| S100A9 | -0.638638665 | 7.262497158 | 1.84E-05 | 7.96E-05 |
| MUC1 | 0.534041963 | 6.223635156 | 2.09E-05 | 8.90E-05 |

**Supplementary TABLE 4** LASSO algorithm screening characteristic genes for DN.

|  | **Gene** | **Coef** |
| --- | --- | --- |
| 1 | CDKN1B | -4.027114699 |
| 2 | GDF15 | -2.666667464 |
| 3 | FKBP5 | -2.506132573 |
| 4 | EGR1 | -2.181325516 |
| 5 | PLA2G4A | 1.977071201 |
| 6 | EXT1 | 1.73007961 |
| 7 | COL4A1 | -1.204883836 |
| 8 | MARCKS | 1.009682902 |
| 9 | CCL2 | 0.878294326 |
| 10 | LPL | -0.553963777 |
| 11 | NUPR1 | -0.55116916 |
| 12 | COMP | 0.502567623 |
| 13 | S100A9 | -0.470964371 |
| 14 | ELOVL4 | -0.449361292 |
| 15 | FOS | -0.441913489 |
| 16 | IGF1 | -0.411570261 |
| 17 | NQO1 | -0.406123629 |
| 18 | KPNA2 | 0.336922834 |
| 19 | PLN | -0.319812021 |
| 20 | COL1A1 | 0.118520923 |
| 21 | RSAD2 | -0.044864551 |

**Supplementary TABLE 5** SVM-RFE algorithm screening characteristic genes for DN.

|  | **FeatureName** | **AvgRank** |
| --- | --- | --- |
| 1 | EGR1 | 1.8 |
| 2 | CDKN1B | 3 |
| 3 | GDF15 | 3.8 |
| 4 | FKBP5 | 4 |
| 5 | PLA2G4A | 6.5 |
| 6 | MARCKS | 10.5 |
| 7 | PLN | 12.3 |
| 8 | NQO1 | 12.7 |
| 9 | CCL2 | 12.7 |
